# Pangenomics of flax fungal parasite *Fusarium oxysporum* f. sp. *lini*

**DOI:** 10.3389/fpls.2024.1383914

**Published:** 2024-05-30

**Authors:** Anton Logachev, Alexander Kanapin, Tatyana Rozhmina, Vladislav Stanin, Mikhail Bankin, Anastasia Samsonova, Ekaterina Orlova, Maria Samsonova

**Affiliations:** ^1^ Mathematical Biology and Bioinformatics Laboratory, Peter the Great St.Petersburg Polytechnic University, Saint Petersburg, Russia; ^2^ Center for Computational Biology, Peter the Great St. Petersburg Polytechnic University, Saint Petersburg, Russia; ^3^ Flax Institute, Federal Research Center for Bast Fiber Crops, Torzhok, Russia

**Keywords:** Fusarium wilt, flax, pangenome, *Fusarium oxysporum*, virulence, effectors, *SIX* genes

## Abstract

To assess the genomic diversity of *Fusarium oxysporum* f. sp. *lini* strains and compile a comprehensive gene repertoire, we constructed a pangenome using 13 isolates from four different clonal lineages, each exhibiting distinct levels of virulence. Syntenic analyses of two selected genomes revealed significant chromosomal rearrangements unique to each genome. A comprehensive examination of both core and accessory pangenome content and diversity points at an open genome state. Additionally, Gene Ontology (GO) enrichment analysis indicated that non-core pangenome genes are associated with pathogen recognition and immune signaling. Furthermore, the *Folini* pansecterome, encompassing secreted proteins critical for fungal pathogenicity, primarily consists of three functional classes: effector proteins, CAZYmes, and proteases. These three classes account for approximately 3.5% of the pangenome. Each functional class within the pansecterome was meticulously annotated and characterized with respect to pangenome category distribution, PFAM domain frequency, and strain virulence assessment. This analysis revealed that highly virulent isolates have specific types of PFAM domains that are exclusive to them. Upon examining the repertoire of *SIX* genes known for virulence in other formae speciales, it was found that all isolates had a similar gene content except for two, which lacked *SIX* genes entirely.

## 1 Introduction

Fusarium wilt of flax is a disease that affects many areas worldwide, caused by a specific type of *Fusarium oxysporum* Schlecht., a fungus in the Ascomycota phylum (Gordon, 2017; Zhang and Ma, 2017). This special form (i.e., forma specialis, f. sp.) of *Fusarium oxysporum* is known to infect only flax (Baayen et al., 2000).

Flax is an excellent source of oil and fiber. Oilseed flax is rich in unsaturated fatty acids, lignins, easily digestible proteins, dietary fiber, vitamins, and minerals. It is also used in the production of paints, resins, printing inks, varnishes, and linoleum. Fiber flax varieties are used as the base material to produce textiles and composites. The Fusarium wilt causes substantial economic damage, as it leads to a reduction in both seed productivity and fiber quality (Kommedahl et al., 1970; Dean et al., 2012; Kumar et al., 2014; Rozhmina et al., 2022).

*Fusarium oxysporum* displays several peculiar features that distinguish it from the other *Fusarium* species. Indeed, the phenomenon of sexual reproduction has not yet been observed in *Fusarium oxysporum* despite the presence of the conserved mating-type genes (either *MAT1–1* or *MAT1–2*) that are typical of sexual species (Yun et al., 2000). On top of this, *Fusarium oxysporum* exhibits a polymorphic lifestyle that varies across genotypes from soil saprophytes to endophytic strains and to specialized parasites capable of infecting plants, as well as animals, including humans (Dean et al., 2012). The lifestyle is mainly set by genetics, however switching between different lifestyles may result from a loss of dispensable chromosomes or be triggered by parasexual processes.

Phytopathogenic *Fusarium oxysporum* strains are usually found in soil as saprophytes. Nonetheless, when environmental conditions are right, they infect plants by penetrating roots and by colonizing vascular tissues (Gordon, 2017). Strains with the same host range are grouped in a forma specialis. Most *Fusarium oxysporum* special forms are restricted to a single plant species, yet some may infect several species, which are either close relatives or at least belong to one family (Kistler, 1997; O’Donnell et al., 1998; Edel-Hermann and Lecomte, 2019). To date, around 100 forms of *Fusarium oxysporum* (Dean et al., 2012) have been identified, with *Fusarium oxysporum* f. sp *lini* (*Folini*) being one of them. The strains of *Folini* have been separated into several clades, implying that its pathogenicity is of a clonal origin (Samsonova et al., 2021).

Recently it has been shown that in *Fusarium oxysporum* strains the estimated genome size varies from 54 Mb to 77 Mb, while the number of chromosomes ranges from 9 to 20 (Ma et al., 2010; Fokkens et al., 2020; Kanapin et al., 2020; Wang et al., 2020). Importantly, all sequenced *Fusarium oxysporum* chromosomes, even those assembled with the Hi-C data (Fokkens et al., 2020; Wang et al., 2020), are predicted pseudomolecules. Only in a handful of them the concordance with physical chromosomes was examined (Ayukawa et al., 2018). The predicted gene count in different *Fusarium oxysporum* genomes vary between 13K and 18K, with the average number of predicted gene models being 17K. Yet, mapping transcriptome sequencing data to *F. oxysporum* f. sp. *lycopersici* genome assembly, built with LRS technology data, predicted 50% more gene models (Sun et al., 2022)

Comparative synteny analysis reveals that *Fusarium oxysporum* genome has a complex structure encompassing conservative (i.e., conservative or core chromosomes) and variable parts (i.e., dispensable or accessory chromosomes). The latter is lineage-specific, meaning that it either exhibits poor syntenic alignment with other *Fusarium oxysporum* genomes from distinct lineages (Ma et al., 2010; Williams et al., 2016; Armitage et al., 2018) or does not align to them at all. The dispensable genome has a low density of genes and a high abundance of transposons. It has a high number of variants and undergoes rapid evolutionary changes. On the other hand, conservative chromosomes have a higher gene density, a lower density of repetitive regions and SNPs. Also, they contain a larger number of genes related to primary metabolism (Armitage et al., 2018; Yang et al., 2020).

The majority of well-studied *Fusarium oxysporum* genomes are characterized with 11 conservative chromosomes, with the number of dispensable chromosomes ranging between 1 and 8 (Kanapin et al., 2020; Wang et al., 2020; Samsonova et al., 2021). Remarkably, the dispensable chromosomes may dissipate with little consequence to the fungus’s survival, but with a measurable detrimental effect on its virulence. Upon re-acquisition of such chromosomes, the virulence of the fungus is likely to increase (Ma et al., 2010). These dispensable chromosomes, also referred to as pathogenic-specific chromosomes, contain specific effector genes that are essential for the successive suppression of the host plant immunity (Armitage et al., 2018). The pathogenic-specific chromosomes were found in several special forms including ff. spp. *lycopersici*, *cepae*, *radicis-cucmerineum* and *fragariae* (van Dam et al., 2017; Li et al., 2020a; Henry et al., 2021; Sakane et al., 2023). Notably, the horizontal transfer of the whole chromosome or its part into a non-pathogenic strain Fo47 have been registered previously for ff. spp. *lycopersici* and *radicis*-*cucumerinum* (Ma et al., 2010; Vlaardingerbroek et al., 2016; van Dam et al., 2017; Li et al., 2020a, Li et al., 2020b). The resulting transformed strains can induce the same disease symptoms as the donor strains on the corresponding host plants. The *Folini* strain MI39 has 11 conservative and 4 dispensable chromosomes, among which chromosomes 12, 13 or 15 are potentially pathogenic-specific chromosomes due to the presence of *SIX* genes encoding specific effectors. However, a focused experiment is required to validate this fact (Kanapin et al., 2020; Samsonova et al., 2021).

The set of genes in the dispensable parts of the individual *Fusarium oxysporum* genomes varies significantly between ff. spp (Plissonneau et al., 2017; Jangir et al., 2021; Sabahi et al., 2021), clonal lineages and surprisingly also within a single f. sp (de Sain and Rep, 2015; van Dam et al., 2016), probably due to their polyphyletic origin (Zhang and Ma, 2017). Capturing the whole set of genes either of *Fusarium oxysporum* or a single f. sp. within *Fusarium oxysporum* is of paramount importance for understanding the mechanisms underlying genome evolution and plasticity, especially for addressing various questions regarding parasite’s adaptation to the host plant, as key determinants of pathogenicity are often functionally redundant and are encoded by genes not shared among all lineages or ff. spp (van Dam et al., 2018; Guallar et al., 2022)

Pangenome research (Badet and Croll, 2020; Badet et al., 2020; Alouane et al., 2021), which refers to the characterization of the complete set of genes of a species or an infra-species taxa, is crucial for pinpointing the virulence factors of fungal isolates. We conducted an analysis of the pangenome of 13 *Folini* isolates that belong to 4 clonal lineages with varying virulence. Our study mainly focused on the functional diversity and content of the pansecretome, a collection of secreted proteins that are crucial for the fungus’s pathogenicity. Notably, our findings indicate that strongly and moderately virulent strains have a different range of PFAM domains in non-core pansecretome groups.

Our research has revealed that all *Folini* strains have the same set of *SIX* genes, with the exception of two strains that had no *SIX* genes at all. This suggests that various fungal isolates possess different virulence factors to counteract plant defense mechanisms, and that while the presence of *SIX* genes is likely a contributing factor to strain virulence, it is not the only factor at play.

## 2 Material and methods

### 2.1 *Folini* strains virulence and VCG

The *Folini* strains under investigation were provided by the Federal Crop Research Institute, city of Torzhok, Tver’ region, Russia. Their virulence was assessed under greenhouse conditions using reference cultivars (Tost 3, Tvertza, A-29) with contrasting susceptibility to Fusarium wilt. Mitcherlich vessels were filled with healthy soil by 2/3 of the height. Next, a pure fungal culture was introduced into each vessel (40 grams per vessel), covered with soil and abundantly watered. The seeds of a reference cultivar were sown on the fifth – sixth day after the inoculation.

The pure culture inoculum of a strain was prepared by growing it on the beer-wort agar-agar medium with subsequent incubation on the oat grain substrate (the grain-to-water ratio of 1 to 1.75). After three, maximum four, weeks’ time, once the substrate had been completely colonized and the macro and microconidia had formed, the pathogen was introduced into the soil.

The severity of the disease in а reference cultivar was evaluated during harvest after the onset of the phase of early yellow ripeness. The DSS (Disease Severity Score) grades ranged from 0 to 3, where 0 stands for a healthy plant, 1 indicates a partial plant browning or stem browning from one side, 2 matches a fully browned plant with bolls, and, finally, 3 corresponds to a fully browned plant that collapses before the formation of bolls. Based on these grades the disease severity index (DSI) was calculated according to the formula generally accepted in the phytopathology field:


$$DSI=\;\frac{\sum ab}{AK}\;100\%$$

where *a* - is the number of plants with the same DSS; *b* – is the estimated DSS; *A* is the total number of plants and *K* is the highest DSS grade (i.e., grade 3). A strain was considered strongly virulent, if DSI value exceeds 50%, moderately virulent, if the DSI value is in the range from 20% to 50%, and, finally, weakly virulent in those cases, where the DSI ≤ 20%. Three flax cultivars representing contrasted disease susceptibility groups, namely highly resistant A-29, moderately resistant Tvertza and the highly susceptible Tost3 were used to classify *Folini* strains into strong, moderate and weakly virulent groups.

Vegetative Compatibility groups are determined in accordance with the adapted method first introduced by Puhalla (Puhalla, 1985). The modifications are as follows: to recover nitrogen-nonutilizing (*nit*) mutants we used PDA supplemented with potassium chlorate (15 g/l) and sucrose (20 g/l) as a chlorate-containing medium.

### 2.2 Genome sequencing, assembly and analysis

Fusarium oxysporum strains F200, F365, F418 were grown in Chapek broth medium (30 g/l sucrose, 2 g/l sodium nitrate, 1 g/l potassium monohydrogen phosphate, 0.5 g/l magnesium sulfate, 0.5 g/l potassium chloride, 0.01 g/l ferrous sulfate). DNA was extracted with NucleoSpin Plant II (Macherey-Nagel, Germany) with the addition of RNAse A. DNA sequencing of the F418 genome was conducted at BGI (Hong Kong, China), with 100x coverage by PacBio RS II System and 30x coverage by Illumina HighSeq. Both the remaining 2 isolates i.e., F200 and F365 were sequenced with 30x coverage using Illumina HighSeq (paired-end reads, 150 bp). Hybrid assembly of the F418 genome was performed in three steps: (1) the initial assembly of PacBio contigs was performed with Canu package (v. 1.4) using the default settings (Koren et al., 2017); (2) further polishing and correction of the assembly was done with Pilon software (v. 1.23) (Walker et al., 2014) using sequencing reads generated by Illumina HighSeq; (3) the guided chromosome-level assembly was constructed with MI39 reference genome using RagTag program (version 2.1) (Alonge et al., 2022).

An assembly of genomes sequenced with short reads (Illumina) only includes two steps: contigs assembly with abyss-pe program from the ABYSS package (version 2.1.5) (Simpson et al., 2009), and reference-guided assembly using MI39 reference genome. To ensure the uniformity of data processing the genomes of previously published strains F329, F324, F282, and F287 (Kanapin et al., 2020) were reassembled with RagTag package (Alonge et al., 2022). Besides the genomes of the strains sequenced in (Dvorianinova et al., 2021) (i.e., F456, F476, F482, F483, F525) and newly sequenced strains F200 and F365 were constructed with abyss-pe and RagTag using MI39 as a reference. The quality of every genome assembly was assessed with the QUAST program (version 5.0.2) (Gurevich et al., 2013). Short reads of 12 *Folini* strains were aligned to the MI39 reference genome using bwa-mem (Li and Durbin, 2009) with default parameters.

To accomplish gene prediction and functional annotation of the proteins, we used BUSCO (v 4.0.4) (Seppey et al., 2019) with parameters “–augustus” and “-l hypocreales_odb10”, respectively. Variant calling for the alignments was completed with NGSEP version 4.0 (Tello et al., 2022) Synteny analysis was done using Satsuma2 program (Grabherr et al., 2010).

Protein orthogroups were identified with silix software (version 1.3.0) (Miele et al., 2011), where the minimum percent of identity to accept blast hits for building families has been set to 0.8. The pangenome accumulation curves were plotted using R vegan package’s specaccum function (Oksanen et al., 2018)

Candidate secreted proteins were identified in three steps. First, secretory signal peptides were predicted using signalP tool (version 5.0) (Nielsen et al., 2019). Then, the TMHMM (v2.0) program was used to keep proteins without a transmembrane domain or with a single transmembrane domain in the N-terminal signal peptide (Krogh et al., 2001). Lastly, PredGPI was applied to remove sequences containing GPI (glycosylphosphatidylinositol) anchors (Pierleoni et al., 2008). Subsequently, the secretomes were screened for CAZymes using a stand-alone version of DBCan2 suite (Zheng et al., 2023) and for effector genes using EffectorP software (version 3.0) (Sperschneider and Dodds, 2022). *SIX* genes were not accounted for in the BUSCO program’s prediction, leading to their inclusion in the final count. Putative peptidases and peptidase inhibitors were predicted using the MEROPS database (Rawlings et al., 2017).

### 2.3 Phylogenetic analysis

For the purposes of phylogenetic analysis, EF-1α gene sequences were selected from 53 strains. These include 13 *Folini* genomes from this study, 20 strains of *Folini* available from the NCBI repositories, and 19 strains of different *F. oxysporum* ff. spp. and *F. solani, F. graminearum, F. verticelloides*, and *F. avenaceum* were used as an outgroup (**Supplementary Table S1**). First, the sequences were aligned with CLC Sequence Viewer tool (http://www.qiagenbioinformatics.com). Next, the maximum likelihood phylogeny tree was constructed with the Tamura-Nei model using 1000 bootstrap replicates, as implemented in the IQ-TREE tool (Minh et al., 2020).

A phylogenetic analysis of *SIX* genes was done using sequences from 13 *Folini* strains and other special forms of *F. oxysporum* (**Supplementary Table S2**). All the sequences were obtained from the NCBI database (https://www.ncbi.nlm.nih.gov/nucleotide/) and aligned with the aforementioned the CLC Sequence Viewer. Again, a consensus tree was built using IQ-TREE (Minh et al., 2020) with bootstrap support (1000 replicates). The following substitution models for each of the trees constructed were in use: *SIX1* -TNe+G4, *SIX7* - K2P+I, *SIX10* - K2P+G4, *SIX12* - K2P, *SIX13* - K2P.

The whole-genome SNP-based phylogeny (as identified in the 12 *Folini* genomes) was inferred by MEGAX software (version 10.1.8) (Tamura et al., 2021), where the MI39 genome served as a reference.

### 2.4 RNA isolation and differential gene expression analysis

Seedlings of the Fusarium wilt-resistant flax cultivar Atalante, as well as the seedlings of the susceptible cultivar LM98, were infected with the fungus strain MI39. RNA isolation from infected and uninfected plants was performed in three independent replicates on days 3 and 5 post-inoculation. Thus, RNA was obtained from 21 samples in total, where each sample includes material collected from three to five roots. Total RNA from infected roots was isolated using the RNeasy Mini Kit (QIAGEN, Germany). The isolated transcripts were sequenced on the DNBSEQ platform at the Beijing Genome Institute (BGI) (paired-end, 100 bp reads). The resulting data was filtered using SOAPnuke (Chen et al., 2017). To assess read abundance for reference transcriptomes of both flax (GCA_000224295.2) and *Folini* (MI39 strain) (Bray et al., 2016) the Kalisto tool (v.0.44.0) with bootstrap support for 100 samples and a k-mer length of 31 nucleotides was applied. Further downstream analysis was conducted using the sleuth package (version 0.30.1) (Pimentel et al., 2016). Transcripts characterized with low coverage values (i.e., 5 reads in either half or more than half of the samples undergoing the comparison) were filtered out and excluded from further consideration.

### 2.5 Analysis of the *SIX* genes

*Fusarium* cultures were grown in Chapek Broth medium for a week in a shaker incubator at 25°C and 100 rpm. Then mycelia were concentrated and rinsed twice in sterile water by centrifugation (3200 rpm) for as long as 15 min at 4°C. Next, the washed mycelia were collected, placed into mortars and dried in a thermostat at 32°C for 24 hours. The product was then powdered with sterile pestles. To extract DNA from the mycelial powder, we used DNeasy Plant Mini Kit (Qiagen) in accordance with the manufacturer’s recommendations. DNA quality was assessed by Qubit 4.0 fluorimeter (Thermo Fisher Scientific) and electrophoresis in 1% agarose gel with subsequent visualization by GelDoc system (Bio-Rad). Finally, we ran PCRs with specific oligonucleotide primers (**Supplementary Table S3**) to evaluate expression levels of *SIX1 — SIX 14* genes in each strain. Likewise, PCR products were documented by electrophoresis in 1% agarose gel using the GelDoc system (Bio-Rad).

Nucleotide sequences of genes *SIX1, SIX7, SIX10, SIX12* and *SIX13* were extracted from Genbank. Homologous loci in *Folini* genomes were identified with the blast+ tool. The resulting homology regions were analyzed with the MIT GENSCAN web server (Burge and Karlin, 1998) and the FindORF tool from the NCBI web server. The predicted polypeptides were aligned to the homologous regions to visualize introns with CLC Sequence Viewer (QIAGEN Aarhus).

## 3 Results

### 3.1 *Folini* strains and genomes

Ten *Folini* strains were isolated from flax straw or from vegetative plants cultivated in the Tver region (Russia). In addition, two isolates, F200 and F456, came from the Netherlands and France correspondingly, while the F365 strain was obtained from China (**Table 1**). Three flax cultivars distinguished by contrasting disease susceptibility, namely A-29 - highly resistant, Tvertza - moderately resistant and, finally, Tost 3 – the highly susceptible type, were used to estimate strain virulence under greenhouse conditions by calculating the Disease Severity Index (DSI) (**Table 2**), where the DSI is a normalized proportion of genotypes with identical disease symptoms (see Section 2.1). Thus, *Folini* strains F282, F287, F324, F329, F483 and MI39 were classified as strongly virulent, while strains F200, F365, F418, F456, and F482 belog to the weak virulence group. Finally, strains F476 and F525 developed a moderate response to infection.

**Table 1:** *Folini* strain characteristics.

Strain	Origin	Virulence	Sequencing	Genome size (Mbp)	Chromosome number	Gene number
MI39	Torzhok distr.	strong	Illumina+PacBio	69,5	20	14 234
F200	the Netherlands	weak	Illumina	64,0	20	14 352
F282	Tver’ distr.	strong	Illumina	64,2	20	14 207
F287	Tver’ distr.	strong	Illumina	64,1	20	14 304
F324	Tver’ distr.	strong	Illumina	65,7	20	14 300
F329	Tver’ distr.	strong	Illumina	64,9	19	14 332
F365	China	weak	Illumina	57,1	20	14 395
F418	Tver’ distr.	weak	Illumina+PacBio	64,9	20	14 200
F456	France	weak	Illumina+NanoPore	55,7	20	14 334
F476	Tver’ distr.	moderate	Illumina+NanoPore	57,9	20	14 260
F482	Tver’ distr.	weak	Illumina+NanoPore	53,2	20	14 466
F483	Tver’ distr.	strong	Illumina+NanoPore	53,7	20	14 087
F525	Tver’ distr.	moderate	Illumina+NanoPore	59,5	20	14 382

**Table 2:** Strain virulence and VCGs.

Strain	DSI, %			VCG
	Tost 3	Tvertza	A-29	
F282	69,9	27,8	12,3	004416
F287	66,6	19,5	16,7	004416
F324	69,9	24,2	15,4	004416
F329	72,7	29,9	8,0	004416
MI39	86,3	25,3	6,5	004416
F200	12,5	0	0	004416
F365	15,6	0	0	004415
F418	18,3	5,6	0	004417
F456	16,7	19,0	0,2	004417
F482	20,0	15,3	1,0	004418
F476	31,4	15,1	6,2	004417
F525	32,0	14,1	14,7	004416
F483	62.4	15,3	12,7	Not available

The ability of the hyphae of two individual fungal isolates to fuse and form viable heterokaryons (i.e., vegetative compatibility) may be considered indirect evidence of genetic similarity (Kistler, 1997; Katan and Primo, 1999). Because of the above, we interrogated 11 *Folini* strains to determine Vegetative Compatibility (VCG) groups (**Table 2**). Of the four VCGs revealed, the largest of them, i.e., group “004416” contains seven strains, including MI39. The next group by size, the “004417”, has three members, while groups “004415” and “004418” are represented by one strain each (F365 and F482, correspondingly).

The MI39 reference genome was constructed from PacBio long reads and was later polished and corrected using short sequencing reads generated by Illumina technology as described in (Kanapin et al., 2020). Out of the 29 scaffolds, 20 scaffolds of the resulting reference genome assembly have been identified as chromosomes based on their homology to the *Fusarium oxysporum* f. sp. *lycopersici* reference genome and the similarity of the genome assemblies of other *F. oxysporum* f. sp. *lini* isolates, as elaborated in (Kanapin et al., 2020). The provisional attribution of the *Folini* chromosomes into two genomic compartments has been facilitated by the syntenic analyses of the MI39 strain that were previously reported (Kanapin et al., 2020; Samsonova et al., 2021). Consequently, chromosomes 1 – 11 and 16 – 19 are assigned to the conservative part, whereas chromosomes 12 –15 and 20 are allocated to the variable compartment.

We used the MI39 genome as a reference to reassemble the genomes of 12 *Folini* strains sequenced by different methods (**Table 1**). The sizes of assembled genomes vary from 53.2 to 69.5 Mbp, with average GC content equal to 48.1%. The N50 value ranges from 28,09 to 173,71 kbp. Both numbers of predicted chromosomes and gene models are similar across thirteen *Folini* genomes, with the latter ranging from 14,087 to 14,395. The majority of the strain genomes are comprised of twenty chromosomes, while F329 has nineteen of them in the predicted karyotype.

**Figure 1:**
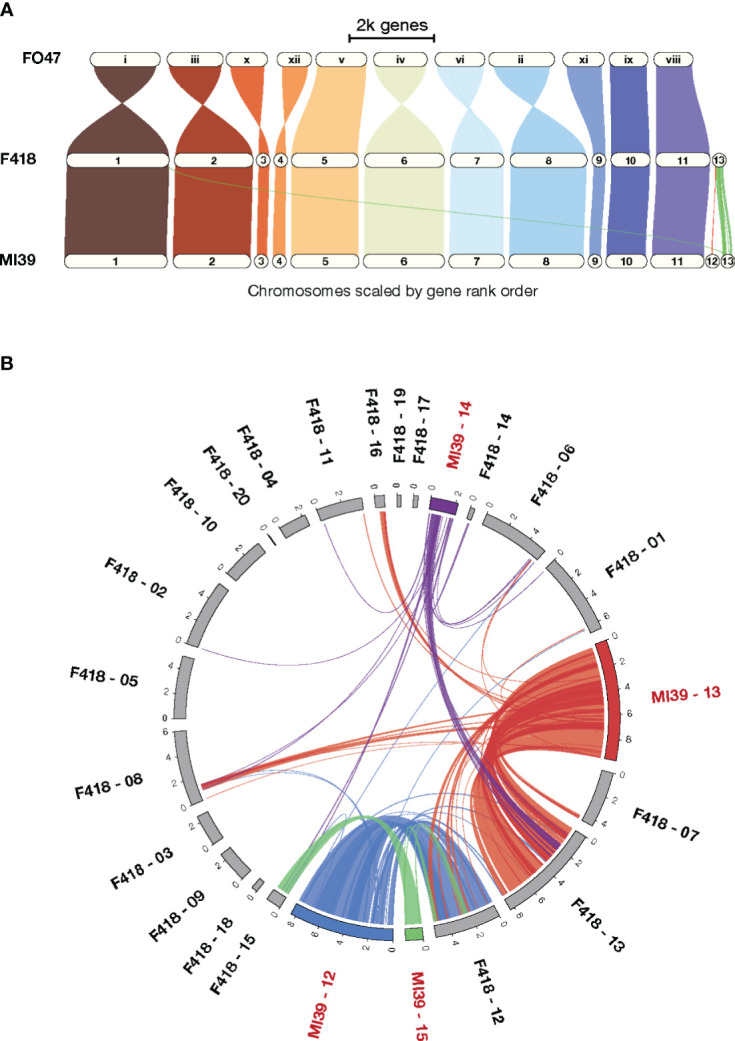
A comparison of the MI39 and F418 genome assemblies. **(A)** The GENESPACE syntenic map (i.e., riparian plot) of syntenic regions in the genome comparison of the *Folini* MI39 and F418 strains and Fo47 endophytic strain. **(B)** Circos plot (Krzywinski et al., 2009) presenting syntenic regions between MI39 and F418 dispensable chromosomes.

To gain insight into patterns of synteny and orthology across *Folini* isolates we compared MI39 and F418 strains as a) their genome sequences resulted from hybrid *de novo* genome assembly, and b) the isolates demonstrate contrasting pathogenic potential. Specifically, MI39 is a highly virulent strain, while F418 exhibit weak virulence. To analyze syntenic patterns, we ran GENESPACE (Lovell et al., 2022) software on MI39 and F418 genome assemblies. The conservative chromosomes of the above strains are similar in size, while the dispensable chromosomes of the MI39 isolate, namely, chromosomes 12–15, differ in length from their counterparts in F418 (**Figures 1A, B**). As expected, the conservative chromosome sets of MI39 and F418 strains demonstrate a high degree of similarity in gene order. Also, the conservation pattern did not change once *F. oxysporum* Fo47 endophytic strain was added to the analysis (Wang et al., 2020). However, we observed at least eight whole chromosome inversions in the Fo47 genome (**Figure 1A**).

The dispensable chromosomes of fungal pathogens are associated with virulence and host-specificity (Frantzeskakis et al., 2019; Torres et al., 2020). Syntenic analysis of dispensable chromosomes in MI39 and F418 isolates revealed chromosomes 15 and 14 as the most and least conserved ones, respectively. Chromosome 15 showed the highest level (64.3%) of similarity in gene order between the aforementioned strains. In contrast, chromosome 14 shares syntenic blocks with dispensable chromosomes 13, 14, and 15 and core chromosomes 1, 6, and 8. It is also longer in MI39 as compared to F418. (**Figure 1B**). Similarly, other accessory chromosomes of MI39 share syntenic blocks with core and non-homologous dispensable chromosomes of F418, as shown in **Figures 1A, B**.

**Figure 2:**
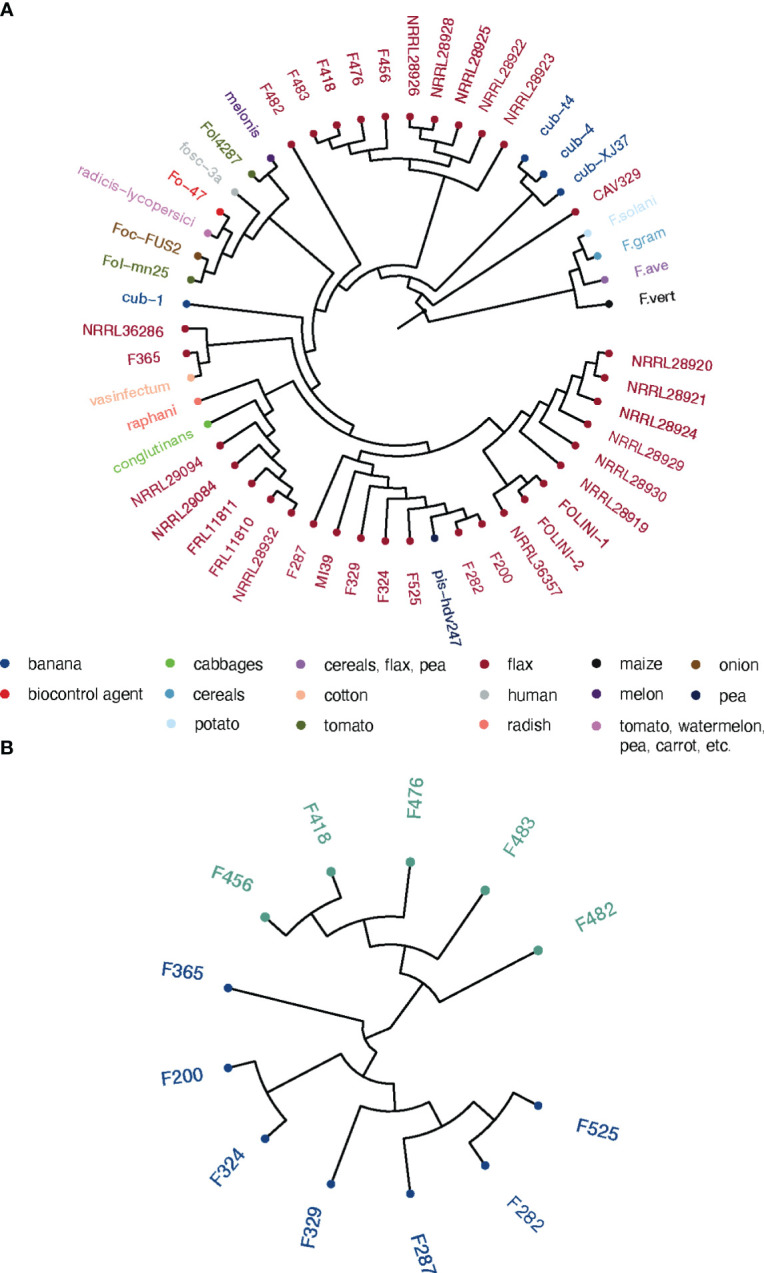
Phylogeny of *Folini* isolates. **(A)** The phylogeny of *F*. *oxysporum* isolates infecting flax and various other hosts as inferred with the maximum likelihood algorithm run on EF-1α gene sequences. **(B)** Neighbor-joining tree for 12 *Folini* genomes generated with whole-genome sets of SNPs as discovered in respective sequences.

We performed the phylogenetic analysis to resolve the relationships and estimate genetic distances between *Folini* strains. Two phylogenetic trees were constructed: (1) a maximum likelihood (ML) tree for EF-1α sequence data for a group of 50 isolates, including 13 *Folini* genomes from this study, 20 *Folini* isolates from NCBI database (https://www.ncbi.nlm.nih.gov/nucleotide/), and 26 different *F. oxysporum* ff. spp. isolates, while 4 other *Fusarium* species were used as an outgroup (**Supplementary Table S1**, **Figure 2A**), and (2) a neighbor-joining (NJ) cladogram based on a whole-genome SNP set for 12 *Folini* strains and MI39 genome as a reference (see **Figure 2B**). On both trees, most of the strains clustered into two distinct clades, while F482 appeared to form a separate branch. It is worth noting that the F365 isolate was in a separate branch on the NJ tree, but grouped together with other isolates obtained from flax and cotton plants on the ML tree, forming a clade.

### 3.2 *Folini* pangenome

**Figure 3:**
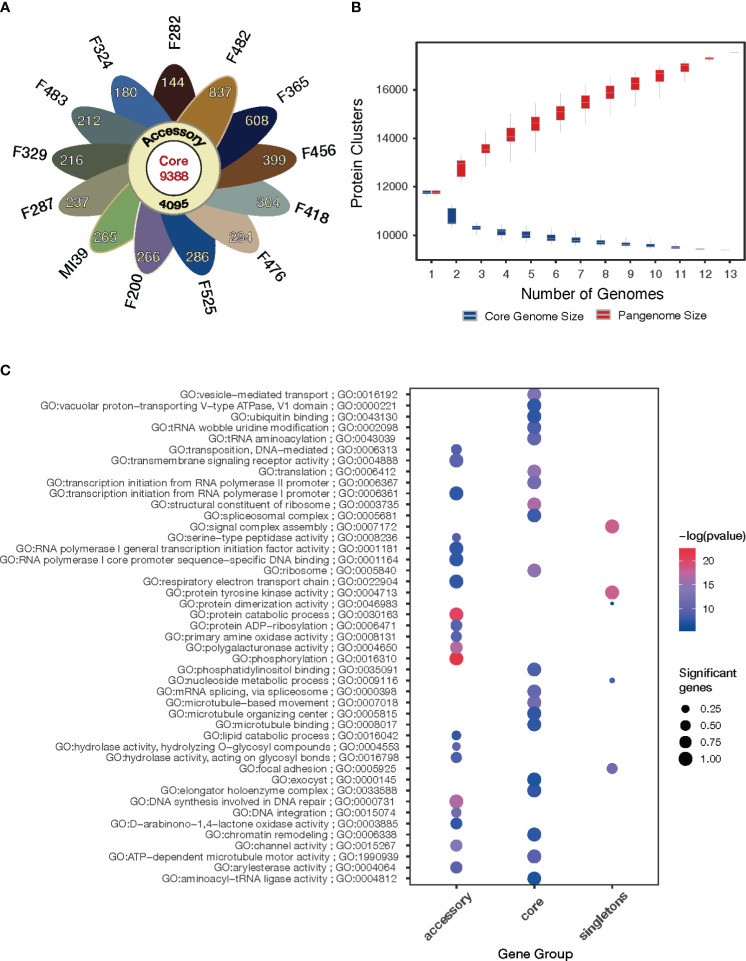
Pangenome of the *Folini* strains. **(A)** The flower plot shows the numbers of core proteins (center), accessory proteins (annulus) and singletons (petals) encoded by the genomes of the 13 *Folini* strains. **(B)** The *Folini* pangenome accumulation curves. **(C)** Functional annotation of core, accessory and singleton proteins.

To estimate the genomic diversity of *Folini* strains and gain insight into its gene repertoire, we constructed a pangenome by clustering 185,853 proteins identified in the 13 *Folini* genomes. We thus detected 17,731 non-redundant orthologous protein clusters (othogroups), of which 9388 (53%) are core orthogroups shared among all genomes (**Figure 3A**). Moreover, 4095 (23%) of all orthogroups are present in some but not all genomes (i.e., accessory orthogroups) and 24% of the orthogroups (4248) are singletons composed of genes found in one of the genomes only. Curiously, F482 and F365 strains have the highest proportion of singletons, 837 and 608 genes, respectively, while the lowest proportion of singletons (144 and 180 genes) is detected in F282 and F324 isolates, respectively. The rest of the strains contain comparable numbers of singletons in the interval sandwiched by 212 and 399 genes.

The gene accumulation curve (**Figure 3B**) demonstrates that the number of core genes continually decreases with the addition of new genomes. On the contrary, pangenome size, defined as a total number of non-redundant orthologous protein clusters, grows steadily with the iterative addition of new genomes, which eventually characterizes the *Folini* genome as open one.

The GO enrichment analysis (FDR< 0.001) of the core, accessory and singleton proteins encoded by the *Folini* pangenome reveals clear differences in their functions (**Figure 3C**). The core proteins are significantly enriched in terms associated with basic cellular and molecular functions, such as microtubule-based movement, ribosome, structural constituent of the ribosome, vesicle-mediated transport, spliceosomal complex, and chromatin remodeling. As expected, the accessory proteins demonstrate enrichment of GO categories linked to pathogenicity and defense mechanisms (e.g. channel activity, D−arabinono−1,4−lactone oxidase activity, hydrolase activity, phosphorylation, polygalacturonase activity, primary amine oxidase activity, protein ADP−ribosylation, serine-type peptidase activity, transmembrane signaling receptor activity), as well as to DNA transposition (e.g. DNA integration, DNA synthesis involved in DNA repair, transposition). Finally, singleton proteins are enriched in terms associated with pathogen recognition and immune signaling, such as cell adhesion, assembly of signal complexes, and protein tyrosine kinase activity.

### 3.3 Pansecretome and virulence

Fungus interaction with the plant immune system, as well as the digestion of plant cells to get nutrients, are accomplished by the secretion of specific proteins that make up an individual secretome (de Sain and Rep, 2015; Jashni et al., 2015; Berlemont, 2017; Kameshwar and Qin, 2018), which, incidentally, is roughly of the same size in different *Folini* strains. The pansecretome was assembled from 621 orthogroups of secreted proteins (3,5% of the *Folini* pangenome). The core and non-core orthogroups of secreted proteins comprise 40,3% and 59,7% of the pansecretome, i.e., 250 and 371 clusters, respectively. Among the non-core orthogroups, 229 clusters (36,9% of the pansecretome) are assigned to accessory orthogroups, while 142 orthogroups (22,6% of the pansecretome) are singletons.

**Figure 4:**
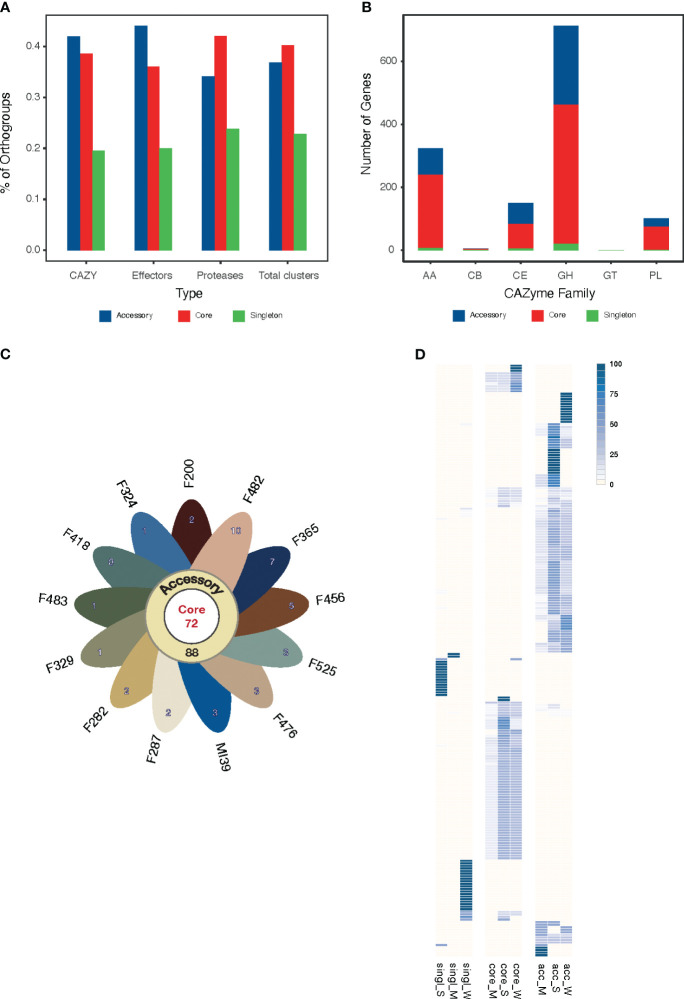
**(A)** The proportion of core, accessory and singleton orthogroups within the pansecretome functional classes. **(B)** Core, accessory and singleton protein numbers within the CAZYme functional classes: AA, auxiliary activities; GH, glycosyl hydrolases; CE, carbohydrate esterases; PL, polysaccharide lyases; GT, glycosyltransferases; CB, carbohydrate-binding modules. **(C)** Flower plot presenting the number of core (center), accessory (annulus) and singleton (petals) orthogroups in the effectome of the 13 *Folini* strains. **(D)** Heatmap of PFAM domain frequencies observed in core (“core”) and non-core (“single” stands for singleton and “acc” stands for accessory) effectome and in strains with moderate (M) and strong (S) virulence. The intensity of the blue color is proportional to the PFAM domain frequency.

The pansectretome primarily comprises three functional groups: CAZYmes, effector proteins, and proteases (see **Table 3**), where the non-core orthogroups dominate within each functional class (**Figure 4A**).

**Table 3:** A number of genes encoding secreted proteins, effectors, proteases and CAZYmes found in the individual *Folini* genomes.

Strain	Number of genes encoding			
	secreted proteins	effectors	proteases	CAZYmes
F200	368	110 + 7*	55	105
F282	350	109 + 7	47	99
F287	359	111 + 7	48	100
F324	372	112 + 7	48	103
F329	345	105 + 7	48	101
F365	356	112	46	94
MI39	340	108 + 8	47	93
F418	342	102 + 7	49	95
F456	346	109 + 7	50	94
F476	347	105 + 6	49	96
F482	367	109	51	112
F483	349	105 + 8	51	98
F525	371	113 + 6	50	102

*The number of *SIX* genes in a strain.

We classified CAZYmes into six groups according to their functions and analyzed their abundance and distribution among pansecretome categories (**Figure 4B**). The core genes dominate in the most abundant classes, which are glycosyl hydrolases (GH), auxiliary activities (AA) and carbohydrate esterases (CE).

The average number of effector proteins identified in individual *Folini* genomes is about 114 (**Table 3**). The *Folini* pansecretome contains 200 effector orthogroups. Of these, 88 are accessory, 40 belong to one isolate only and 72 are conserved between all strains (**Figure 4C**). The number of singletons agrees with the phylogenomic relationship between strains. For example, the F482 strain contains the maximum number of singletons (10 orthogroups), while the F365 strain and F456 isolate incorporate 7 and 5 singletons correspondingly. The number of singleton effectors in the remaining ten genomes ranges between 3 and 1.

The *Folini* genome has dual compartmentalization encompassing conserved and dispensable (i.e., lineage-specific) parts. Incidentally, the reference MI39 strain has 116 effector genes (**Table 3**; **Supplementary Table S6**), of which 63 and 53 genes belong to core and accessory pansecretome, respectively. F418, the second strain sequenced with LRS technology, has the same number of core effectors and 7 non-core effectors fewer than MI39. The majority of genes encoding these proteins are located on conserved chromosomes, while only 12 and 9 non-core effector genes map to dispensable chromosomes of MI39 and F418 correspondingly.

The expression level of predicted effectors in MI39 mycelia was assessed in both liquid culture and *in planta* by observing the infection progress in seedlings from resistant (Atalante) and susceptible (LM98) flax varieties over time. While all MI39 effectors were expressed, their levels varied. One-third of the effectors showed low expression in both mycelium and infected plants. The remaining effectors are divided into two categories: (1) those present in both the mycelium and the plant and (2) those only found in the plant (as shown in **Supplementary Figure S1**). The effector orthogroups’ presence-absence heatmap in *Folini* strains indicates a clear separation between strongly and weakly virulent strains (**Figure 4D**). Notably, an orthogroup known as FOL004461, consists of eight proteins expressed only *in planta*. Of these proteins, five are present in MI39, as well as in other strongly virulent strains, while two proteins are found in weakly virulent strains, namely F365 and F200, and one is present in the medium virulent strain, F525. These proteins contain three specific PFAM domains: MANEC (PF07502), PAN_3 (PF08277), and PAN_4 (PF14295). PAN domains are ubiquitous in diverse proteins and are involved in protein-protein and protein-carbohydrate interactions (Tordai et al., 1999), while the MANEC domain may play a pivotal role in the formation of protein complexes that contain various protease activators and inhibitors (Guo et al., 2004).

We performed a genome-wide annotation of CAZYmes, effector proteins, and proteases using the PFAM database (Mistry et al., 2020). The results of hierarchical clustering of PFAM domain frequencies computed for strongly and moderately virulent *Folini* isolates in core, accessory and singleton categories highlight a remarkable difference in domain repertoire (**Figure 4D**; **Supplementary Figure S2**). It is noteworthy that certain PFAM domains are exclusive to specific virulence groups, with most of these domains being encoded by non-core genes. Among strongly virulent strains, the number of unique PFAMs found in CAZYmes and proteases is three times less than in weakly virulent strains (23 and 68 domains, respectively), as presented in **Supplementary Table S4**. This disparity can be attributed to the close genetic relationship of strongly virulent strains, as evident from most of them being grouped in one clade of the phylogenetic tree (**Figure 2A**). Despite the significant genetic similarity between strongly and weakly virulent strains, the number of unique PFAM domains found in effectors is similar for both, with 26 and 35, respectively (see **Supplementary Table S5**). The PFAM domains found in CAZYmes of strongly virulent strains are commonly found in transmembrane and extracellular proteins, as well as proteins participating in glycoside bond hydrolysis (PF01120, PF07477, PF03632, PF17851, etc.), carbohydrate synthesis (PF05691), and fatty acid metabolism (PF02737). Additionally, weakly virulent strains also possess a functionally similar set of specific domains for CAZYmes, but with the added presence of domains characteristic of methyltransferases (e.g., PF08241, PF13489, PF13649) and host-pathogen recognition (PF13517). The functional diversity of PFAM domains found in effector proteins is particularly high (**Supplementary Table S5**), with strongly virulent strains possessing domains for proteins involved in signaling (PF07645, PF13923) and regulation of transcription (PF05086), while weakly virulent strains exhibit protein domains involved in tyrosine and phenylalanine biosynthesis (PF01817) and chromatin remodeling (PF09011).

### 3.4 *SIX* gene analysis

The pathogen’s virulence is modulated by cysteine-rich small effector proteins called Secreted in Xylem (SIX) proteins (Rep et al., 2004; Houterman et al., 2009; Kashiwa et al., 2013; Schmidt et al., 2013; Ma et al., 2015; van Dam et al., 2017; Widinugraheni et al., 2018). SIX proteins were first discovered in xylem sap of tomato plants (Houterman et al., 2007; Schmidt et al., 2013). The coding of these proteins is attributed to *SIX* genes belonging to 14 gene families and located on pathogenic-specific chromosomes. Nonetheless, formae speciales have diverse SIX protein profiles and gene sequences (de Sain and Rep, 2015; Jangir et al., 2021). Thus, *SIX* genes can be utilized as markers for identifying pathogens.

Surprisingly, we could not find *SIX* genes in two weakly virulent strains (i.e., F365 and F482). Yet, the rest of the 11 *Folini* genomes considered in this study exhibit identical sets of the *SIX* gene families, namely *SIX1, SIX7, SIX10, SIX1*2 and *SIX13*, thus suggesting a conservation of the *SIX* gene repertoire (**Table 4**; **Supplementary Figures S3**, **S7**-**S20**).

**Table 4:** *SIX* gene repertoires identified in each of the 13 *Folini* genomes.

Strain	Chromosome	*SIX1*	*SIX7*		*SIX10*	*SIX12*		*SIX13*
			a	b		a	b	
M39	12	+	+ (x3)	–	+	+	–	+
	13	–	–	–	–	–	+	–
	15	–	–	+	–	–	–	–
F200	12	+	+	–	+	–	–	+
	13	–	–	–	–	–	+ (x3)	–
F282, F287, F324, F329	12	+	+	–	+	+	–	+
	13	–	–	–	+	–	+	–
F365		–	–	–	–	–	–	–
F418	12	+	+	–	+	+	–	+
	13	–	–	–	–	–	+	–
	15	–	–	+	–	–	–	–
F456	12	+	+	–	+	_	–	–
	13	–	–	–	–	+ (x2)	–	–
	15	–	–	+	–	_	–	–
F476	12	+	+	–	+	–	–	+
	13	–	–	–	–	–	+	–
	15	–	–	+	–	–	–	–
F482		–	–	–	–	–	–	–
F483	12	+	+	–	+	+	–	+
	13	–	–	–	–	+ (x2)	–	–
	15	–	–	+	–	–	–	–
F525	12	+	+	–	+	+	–	+
	15	–	–	+	–	–	–	–

“+” and “-” indicate gene presence or absence, (xn) shows the number of copies.

The *SIX1* gene sequence is identical in all genomes except for F418 and F456. The Six1 protein in the F418 isolate has 12 amino acid substitutions, of which one, at position 238 (glutamate to glutamine), is also present in the Six1 protein of the F456 isolate. Also, there are two paralogs of the *SIX7* gene *(i.e., SIX7a* and *SIX7b)* on chromosomes 12 and 15, respectively (**Table 4**). Interestingly, all *Folini SIX*-containing strains have *SIX7a*, while the MI39 genome has three copies of this gene. We found *SIX7b* gene in genomes of the MI39, F418, F456, F476, F483 and F525 strains. The amino acid sequences of *SIX7a* and *SIX7b* are 82% identical. The *SIX10* gene is situated on chromosome 12 and includes one intron. It encodes two polypeptide variants, one with arginine and the other with glutamine at the C-terminus. Two paralogs of the *SIX12* gene, *SIX12a* and *SIX12b*, are present, with widely varying location and number of copies. Most *Folini* strains, M39, F282, F287, F324, F329 and F418 contain both paralogs. In all strains, except for F456 and F483, *SIX12a* maps to chromosome 12, while *SIX12b* is found on chromosome 13. In the F456 strain, two copies of *SIX12a* are located on chromosome 13, while in the F483, there are two *SIX12a* copies on chromosome 13 and only one copy on chromosome 12. In the F476 and the F525 strains, genes *SIX12b* and *SIX12a* are found on chromosomes 13 and 12, respectively (**Table 4**). The F200 strain has three copies of the *SIX12b* gene, all of which are located on chromosome 13. The Six12a and Six12b proteins are almost identical, differing only in two positions: 83 and 91. At these positions, Six12a has glutamate and lysine, while Six12b has lysine and asparagine, respectively. The *SIX13* gene has two introns and is always present as a single copy on chromosome 12 (as shown in **Table 4**). This gene is strongly conserved across strains.

**Figure 5:**
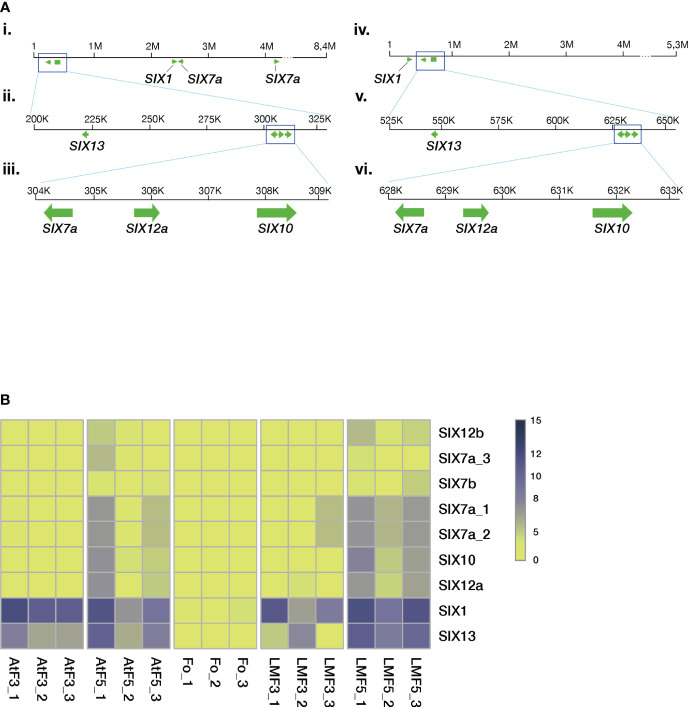
The *Folini SIX* genes. **(A)**. *SIX* gene mapping to chromosome 12 of MI39 (panels i, ii, iii) and F418 genomes (panels iv, v, vi) assembled with the long-read sequencing technology. Note the similarity of the *SIX7a-SIX12a-SIX10* gene cluster between the genomes and the positioning of the *SIX13* gene. **(B)** Heatmap presenting normalized *SIX* gene expression levels in different genomes, where *Fo* samples correspond to mycelia in liquid culture, AtF stands for Atalante flax cultivar (resistant to infection) on the third and the fifth day post-inoculation, and, finally, LMF shows LM98 flax variety (susceptible) on the third and the fifth day post-inoculation. All experiments were carried out in three biological replicates.

We characterized the relative location of *SIX* genes in chromosome 12 using the genomes assembled with the LRS technology (M39 and F418 isolates, **Figure 5A**). The analyses revealed several gene clusters of similar architecture.

Recent results show that *SIX1* and *SIX13* gene families demonstrate host-related clustering, as revealed by phylogenetic analyses (Samsonova et al., 2021). Given these findings, we examined the remaining *SIX* genes to check if the aforementioned host-related grouping holds. The *SIX* gene dataset included members from different *F. oxysporum* ff. spp. alongside three other *Folini* isolates from Australia and Canada (**Supplementary Table S3**). The phylogeny analyses conducted on the *SIX7*, *SIX10* and *SIX12* genes demonstrate that the *Folini* isolates are separate from other ff. spp. (see **Supplementary Figures S4**, **S5**). The phylogeny of the *SIX7* (**Supplementary Figure S4A**) and *SIX12* (**Supplementary Figure S5**) gene families distinctly highlights the difference between *SIX7a* and *SIX7b*, as well as *SIX12a* and *SIX12b* sequence variants, since they occupy separate clades on the trees. We thus conclude that the *SIX7* and *SIX12* gene homologs are of polyphyletic origin. In contrast, as demonstrated in the respective phylogenetic tree, the origin of *SIX10* is monophyletic.

We used RNAseq technology to assess the expression levels of *SIX* genes during the 5-day infection process of flax plants. We conducted this evaluation on seedlings from both resistant (Atalante) and susceptible (LM98) varieties, as shown in **Figure 5B**. *SIX1* and *SIX13* exhibit higher expression levels than other *SIX* genes. *SIX13* expression in LM98 exhibits variable levels of activity over replicates. In contrast to the Atalante variety, in LM98, it is slightly lower and reaches high levels only by the fifth day. The remaining *SIX* genes (and their copies) are either expressed at low or moderate levels. Interestingly, the latter is detected in two copies of *SIX7a*, a copy of *SIX12* and *SIX10* on day five post-infection in the susceptible flax cultivar.

Thus the characterization of the *SIX* gene diversity in *Folini* strains revealed the conservation of their repertoire: most strains had the same set of *SIX* genes regardless of their virulence status. However two *Folin*i isolates lacked any of the *SIX* genes in spite of weak virulence.

### 3.5 Mating-type idiomorphs

It is widely accepted that *F. oxysporum* is a species that reproduces asexually (Gordon and Martyn, 1997). However, the fungus contains a conserved set of genes involved in sexual development and reproduction in other fungi, including mating-type idiomorphs (Yun et al., 2000). We found that twelve of thirteen *Folini* strains have *MAT1–2* idiomorph, while only the F365 strain is *MAT1–1* positive (**Supplementary Figure S6**). In all strains considered, the *MAT1–2* locus contains two genes oriented in opposite directions at its ends. The first gene is *MAT1–2-1*, highly conserved across Sordariomycetes. The second gene encodes a protein exhibiting a high level of homology with Mat1–2-3 protein from *F. avenaceum* and hence was identified as *MAT1–2-3* (Lysøe et al., 2014) The F482 strain has the truncated version of this gene, *MAT1–2-3m*, which lacks the whole third exon and part of the second intron. PCR analysis revealed that *MAT1–2-3* and *MAT1–2-3m* transcripts in liquid mycelial cultures of MI39 were of the expected gene length.

The F365 strain’s *MAT1–1* locus conforms to the architecture characteristic of heterothallic Sordariomycetes (**Supplementary Figure S6**), apart from the fact that the *MAT1–1-3* gene lacks an intron.

## 4 Discussion

In this study, we created a pangenome for 13 isolates of *F. oxysporum* f. sp. *lini (Folini)* at the intra-species level. An average *Folini* genome contains 14,296 genes, contrary to our baseline assessment of the gene repertoire in the pangenome of 17,731 genes. The number of genes found in the genomes of all isolates considered in this study is even smaller – 9,388, which amounts to 54% of the pangenome gene repertoire. Recently, it has been shown that the pangenome of *F. graminearum* is made up of 20,807 non-redundant sequences from 20 isolates. Out of these, 11,560 genes (56%) are non-core genes. Also, the genome of an isolate varies between 12,729 and 13,316 predicted genes (Alouane et al., 2021). According to a study, the *Z. tritici* pangenome constructed with 19 genomes has about 15,474 orthogroups. However, only 60% of these orthogroups (around 9,193) are present in all studied isolates. Additionally, each genome has a gene count ranging between 11,657 and 12,787 (Badet et al., 2020). Despite having a larger number of individual genes, which is an average of 20,645, and a larger pangenome size of 34,639 genes (Fayyaz et al., 2023), the pangenome of 99 F*. oxysporum* f. sp. *ciceris* strains still contains a similar number of core genes as 10,435. Given these findings, the number of core genes in the *Folini* genome stands in the same range as in the genomes of *F. oxysporum* f. sp. *ciceris*, *F. graminearum* and *Z. tritici* (10,435, 9,388 and 9,193, respectively). Most notably, as the analyses of enriched GO terms clearly demonstrate (Alouane et al., 2021; Fayyaz et al., 2023), proteins attributed to the core part of the above pangenomes are associated with primary metabolism, which points to the conservation of essential gene repertoire in genomes of phytopathogenic mycelial fungi.

Our results demonstrate that the *Folini* pangenome is open and has not yet captured the overall diversity of the f. sp. At present, the *Folini* pangenome captures gene content from 4 clonal lineages. Yet, a survey of vegetative compatibility conducted on 74 isolates in wilt nurseries in western Canada identified 12 vegetative compatibility groups (Mpofu and Rashid, 2001), thus emphasizing the need for further research, including sequencing of new strains from different regions.

The *Folini* pangenome contains a relatively high proportion of accessory and singleton genes (46%), indicating the presence of considerable genomic diversity within individual isolates. This observation is further supported by the syntenic analyses of the genomes of MI39 and F418, which revealed significant genomic rearrangements in dispensable chromosomes, as shown in **Figure 1B**. The spectrum of enriched GO terms commonly associated with non-core *Folini* genes highlights their involvement in processes concerned with plant immunity and pathogenicity (**Figure 3C**). It is possible to hypothesize that the virulence potential and adaptation of individual strains to the environment is upheld by a high level of genetic variability that is intrinsic to them. It’s worth noting that the conjecture is strongly supported by the fact that PFAM domains specific to virulence groups are predominantly encoded by non-core genes (as shown in **Figure 4D**; **Supplementary Figure S2**, **Supplementary Table S4**).

The genomes of filamentous plant pathogens are divided into two parts - conservative and dispensable, which creates a concept called the “two-speed genome” (Raffaele et al., 2010; Torres et al., 2020). This genome compartmentalization speeds up the evolution of genomic loci on variable chromosomes, which is crucial for virulence. It’s worth noting that only a small percentage (0.2%) of core genes are found in the dispensable part of both MI39 and F418 genomes. Meanwhile, the proportion of non-core genes located on these chromosomes is much larger (namely, 23.5% in MI39 and 15.5% in F418 - as shown in **Supplementary Table S6**). Despite this, most of the genes that encode the preponderate pansecretome classes - CAZYmes, effectors, and proteases are situated in the conservative part of both genomes and depending on the functional category, between 20 and 40 percent of these genes are non-core.

The fungus pansecretome is an indispensable player shaping host-pathogen interactions (de Sain and Rep, 2015; Jashni et al., 2015; Berlemont, 2017; Kameshwar and Qin, 2018). The *Folini* pansecretome amounts to 3.5% of pangenome and is almost exclusively made of CAZYmes, effector proteins and proteases (**Table 3**), which are distributed nonuniformly over core and non-core orthogroups. Glycosyl hydrolases (GH), auxiliary activities (AA), and carbohydrate esterases (CE) (**Figure 4B**) are abundant classes in CAZYmes. They are involved in cellulose, hemicelluloses, pectin and lignin degradation and are essential for successful invasion and pathogenesis (Berlemont, 2017; Kameshwar and Qin, 2018). Incidentally, the prevalence of the same functional classes over other types of enzymes was also found in ff. spp. infecting legumes (Roy et al., 2020)

To establish a stable infection, fungi secrete effector proteins that suppress the host’s immune response or manipulate the host’s cell physiology (Presti et al., 2015). Somewhat surprisingly, despite their crucial role in understanding the mechanisms driving fungal invasion, the majority of *Folini* effector proteins are still uncharacterized (Jangir et al., 2021). At present, 14 families of SIX proteins have been identified (Does et al., 2008; Schmidt et al., 2013). Most importantly, the repertoire of SIX proteins is diverse in different ff. spp (van Dam et al., 2016). A selected number of SIX proteins found in *F. oxysporum* ff. spp. *lycopersici*, *cubensis* and *conglutinans* were experimentally shown to enhance virulence and are, therefore, genuine effectors (Rep et al., 2004; Houterman et al., 2009; Kashiwa et al., 2013; Ma et al., 2015; Li et al., 2016; van Dam et al., 2017; Widinugraheni et al., 2018). An in-depth analysis of the *Folini SIX* genes revealed the conservation of their repertoire since all strains except two isolates contain an identical set of five gene families, namely, *SIX1, SIX7, SIX10, SIX1*2 and *SIX13* (**Table 4**; **Supplementary Figure S3**). Notably, multiple copies of *SIX7* and *SIX12*, along with paralogous genes, exist in various isolates. Furthermore, when infecting flax cultivars with the MI39 strain, we observed *in planta* expression for nearly all *SIX* genes (**Figure 5B**). Interestingly, the two mildly virulent strains, F365 and F482, lacked any of the *SIX* genes (**Tables 2**, **4**). This implies that *SIX* genes are not always present in pathogenic *F. oxysporum* strains. Likewise, the absence of *SIX* genes was reported in two weakly virulent isolates of *F. oxysporum* f. sp. *cepae* (Taylor et al., 2016), in the genomes of endophytic isolates of *F. oxysporum* f. sp. *ciceris* (Fayyaz et al., 2023) and the *F. oxysporum* isolates obtained from the diseased pea plants collected from the fields in the UK (Jenkins et al., 2021). Also, a low frequency of *SIX* genes is observed in natural populations of *F. oxysporum* isolates sampled across the Australian continent (Rocha et al., 2016). It is possible that the absence of *SIX* genes in *Folini* strains is a result of their distinct origin. However, this seems unlikely as F365 belongs to the same clade as the NRRL36286 that infects flax and the *F. oxysporum* f. sp. *vasinfectum* isolate that infects cotton (as shown in **Figure 2A**). Also, no correlation between *SIX* gene presence and virulence was reported for endophytic isolates of *F. oxysporum* f. sp. *ciceris* (Fayyaz et al., 2023). Thus *F. oxysporum* isolates can be pathogenic in the absence of *SIX* genes, which puts a high priority on analyzing other effectors as potential virulence factors.

*F. oxysporum* is an exclusively asexual species, though it contains a conserved set of mating-type idiomorphs (Yun et al., 2000; Gordon, 2017). We found that 12 of 13 *Folini* strains have *MAT1–2* idiomorph, while F365 is the only *MAT1–1* positive one (**Supplementary Figure S6**). The predominance of *MAT1–2* idiomorph could be due to a geographic factor, as the majority of the strains come from the Tver region in Russia. In contrast to *F. oxysporum* ff. spp. *lycopersici* and *ciceris*, all *MAT1–2 Folini* strains contain additional gene *MAT1–2-3*. Conservation of *MAT* idiomorphs in *F. oxysporum* goes hand in hand with the conservation of other genes required for sexual reproduction (Yun et al., 2000; Zhang et al., 2020). In this context, the evolutionary stability of a functional sex pheromone synthesis and perception pathway is of particular interest (Turrà et al., 2015).

Our study focuses on the gene repertoire of *F. oxysporum* f. sp. *lini*, shedding new light on its genomic diversity. We discovered significant chromosomal rearrangements that are specific to individual genomes. Moreover, our study provides strong support for the hypothesis that *F. oxysporum* isolates utilize different virulence factors during crop infection, allowing them to evade plant defense mechanisms efficiently. Most importantly, our findings clearly demonstrate no relationship between the *SIX* gene content and virulence, which enhances our understanding of the role of *SIX* genes in pathogenicity. However, analysis of the core and accessory pan-genome gene repertoire of *Folini* indicates the open state of the genome and highlights the importance of further research that includes sequencing of new diverse strains from different regions. Charting the *Folini* pangenome blueprint and studying its diversity is an essential and imperative initial step towards uncovering the mechanisms that underlie plant-fungal interactions, and devising new strategies for controlling diseases.

## Data availability statement

The datasets presented in this study can be found in online repositories. The names of the repository/repositories and accession number(s) can be found below: BioProject, PRJNA630722 and PRJNA721899.

## Author contributions

AL: Investigation, Writing – original draft. AK: Conceptualization, Formal Analysis, Methodology, Validation, Writing – review & editing. TR: Funding acquisition, Resources, Writing – original draft. VS: Validation, Writing – original draft. MB: Data curation, Writing – original draft. AS: Software, Visualization, Writing – review & editing. EO: Project administration, Writing – original draft. MS: Writing – review & editing, Supervision, Conceptualization, Methodology.
