# Supplementary material for: Pangenomics of flax fungal parasite Fusarium oxysporum f. sp. lini
Source: Front Plant Sci. 2024 May 30;15:1383914. doi: 10.3389/fpls.2024.1383914 (PMC11169931; doi:10.3389/fpls.2024.1383914)
Supplement: Supplementary file 1 [file DataSheet_1.docx]

Supplementary Material

Pangenomics of Flax Fungal Parasite *Fusarium oxysporum* f. sp. *lini*

Anton Logachev, Alexander Kanapin, Tatyana Rozhmina, Vladislav Stanin, Mikhail Bankin, Anastasia Samsonova, Ekaterina Orlova and Maria Samsonova*

*** Correspondence:** Maria Samsonova: m.samsonova@spbstu.ru

# Supplementary Figures

**Supplementary Figure 1**. Heatmap presenting normalized effector gene expression levels in different genomes, where *Fo* corresponds to mycelia in liquid culture, AtF stands for Atalante flax cultivar (resistant to infection) on the third and the fifth day post-inoculation, and, finally, LMF shows LM98 flax variety (susceptible) on the third and the fifth day post-inoculation. All experiments were carried out in three biological replicates.

**Supplementary Figure 2.** Heatmap of PFAM domain frequencies observed in core and non-core genes encoding CAZYmes (A) and proteases (B) in strains with weak (W), moderate (M) and strong (S) virulence. The intensity of the blue colour is proportional to the PFAM domain frequency. Singlеtons (singl) and accessory (acc) genes make up the non-core part of the pangenome.


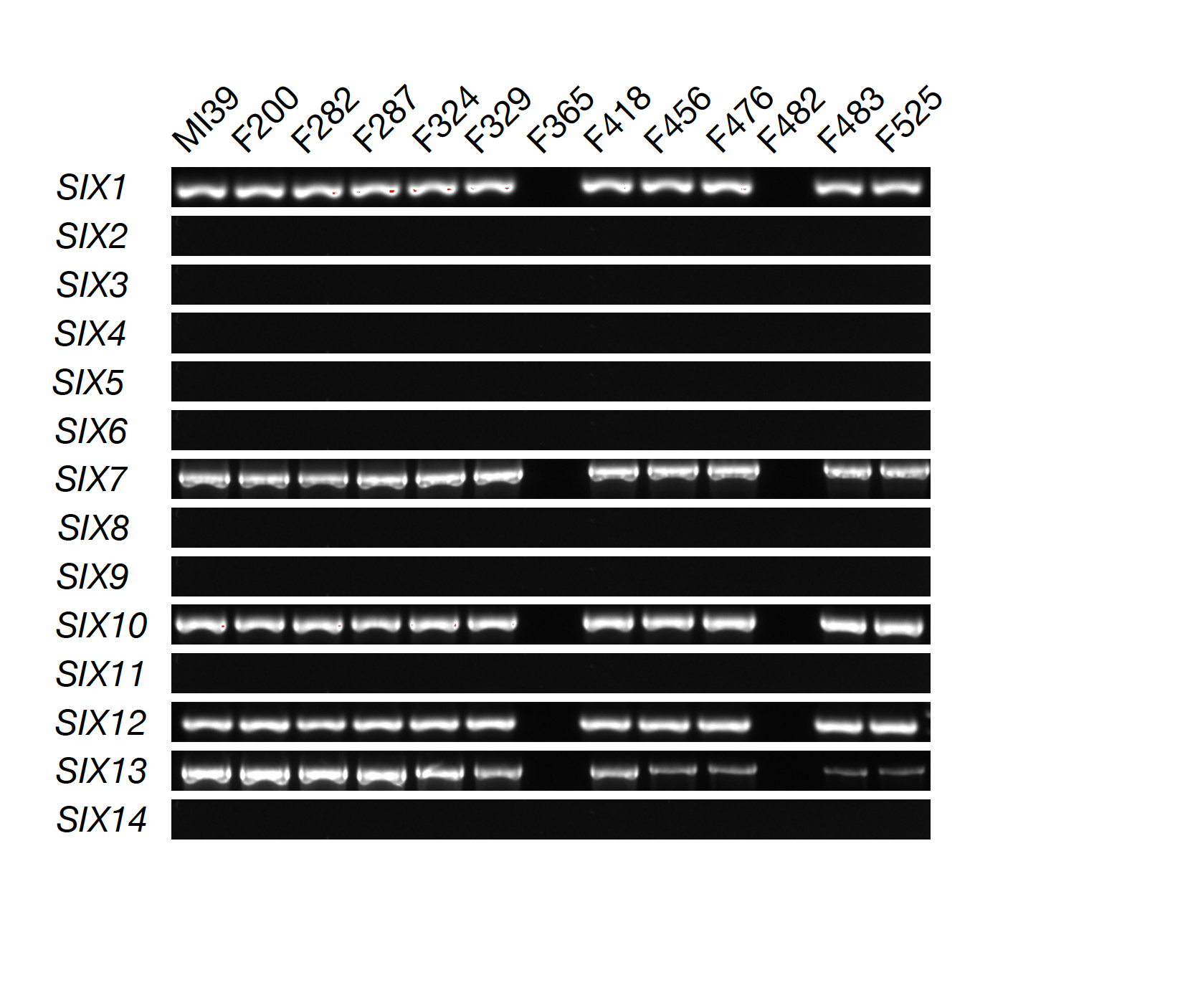


**Supplementary Figure 3.** PCR analyses showing the presence of *SIX* genes in *Folini* isolates. Supplemental Figures 7 – 20 present scans of original gels.

**Supplementary Figure 4.** Consensus trees from the Bayesian phylogenetic analysis of *SIX* genes from the *Fusarium* clade. Tree tips and label colors reflect the pathogen’s host organism. The list of hosts is shown separately. (A) *SIX7* consensus tree, (B) *SIX10* consensus tree.


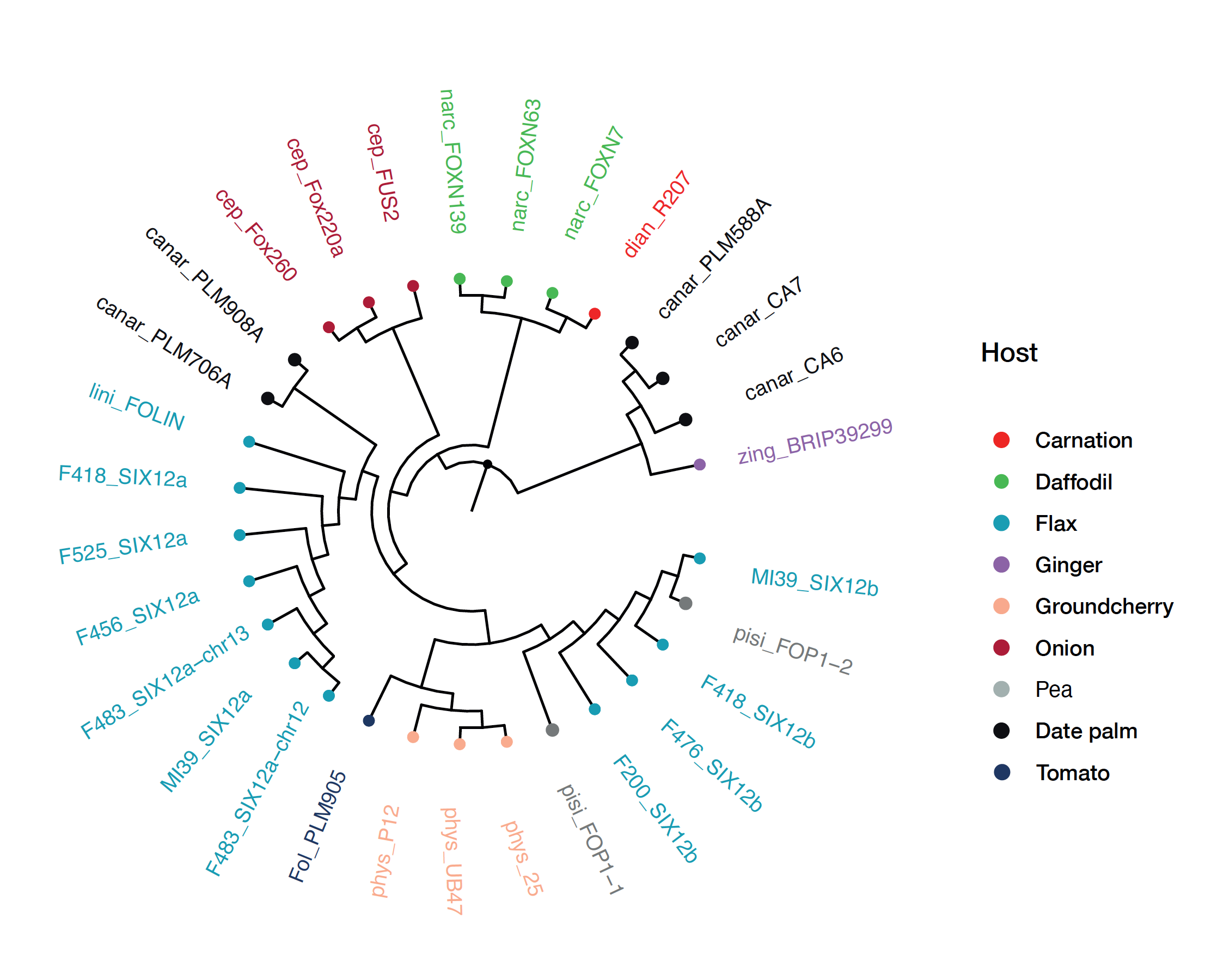


**Supplementary Figure 5.** Consensus tree from the Bayesian phylogenetic analysis of *SIX12* gene from the *Fusarium* clade. Tree tips and label colors reflect the pathogen’s host organism. The list of hosts is shown separately.

**
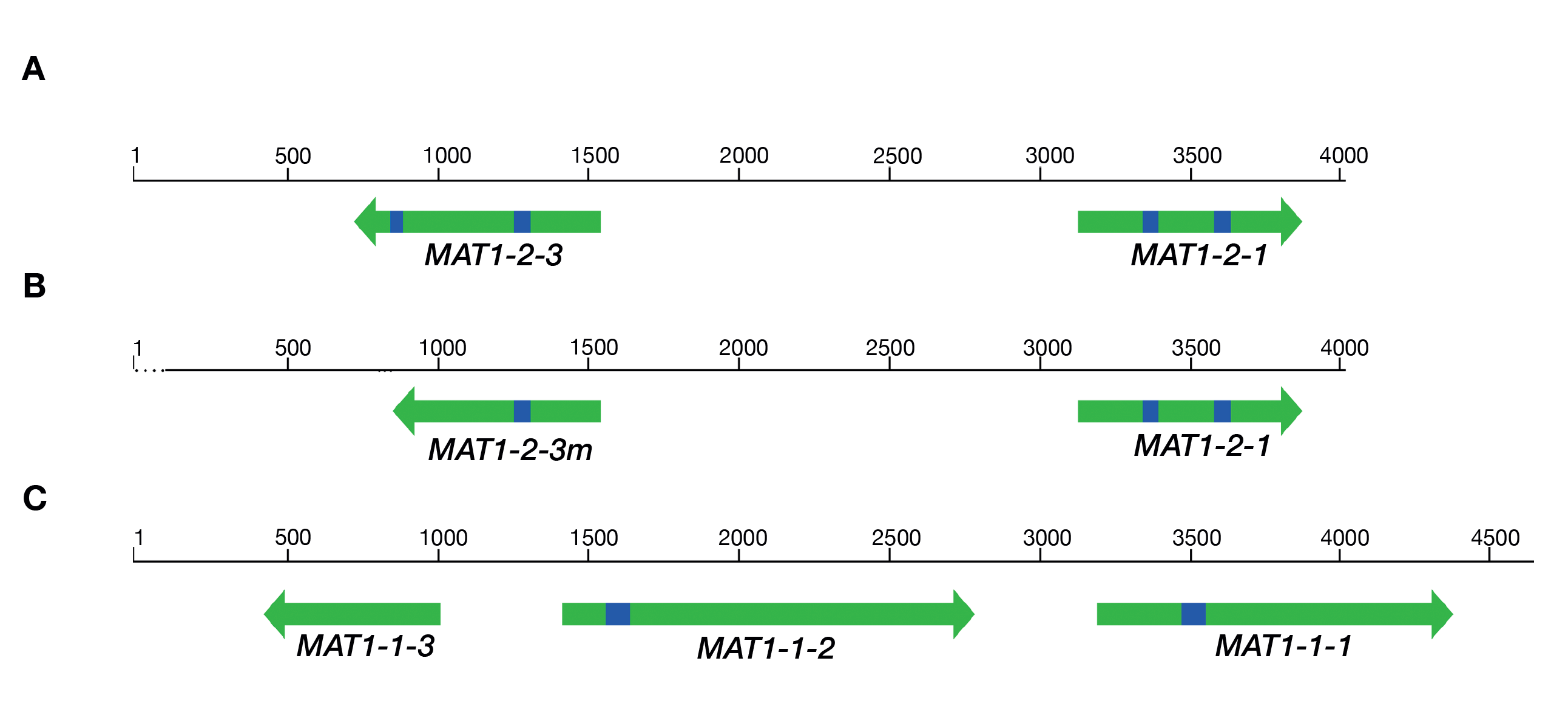
**

**Supplementary Figure 6.** Gene structure of mating type idiomorphs in the *Folini* stains. Structural organization of *MAT1-2* idiomorph in all stains (A) except of F482 (B). (C) The architecture of *MAT1-1* locus in F365 strain.


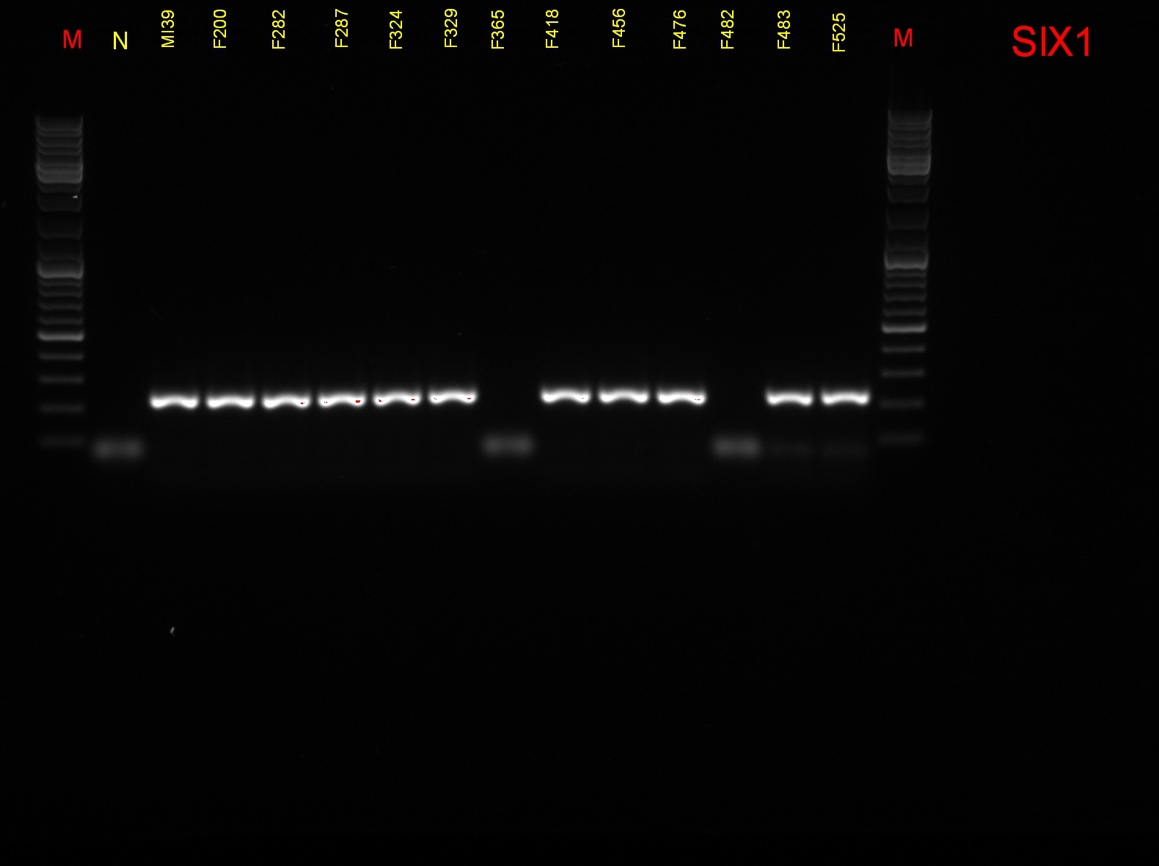


**Supplementary Figure 7.** Scan of the gel showing the presence of *SIX1* gene in the *Folini* isolates**.**


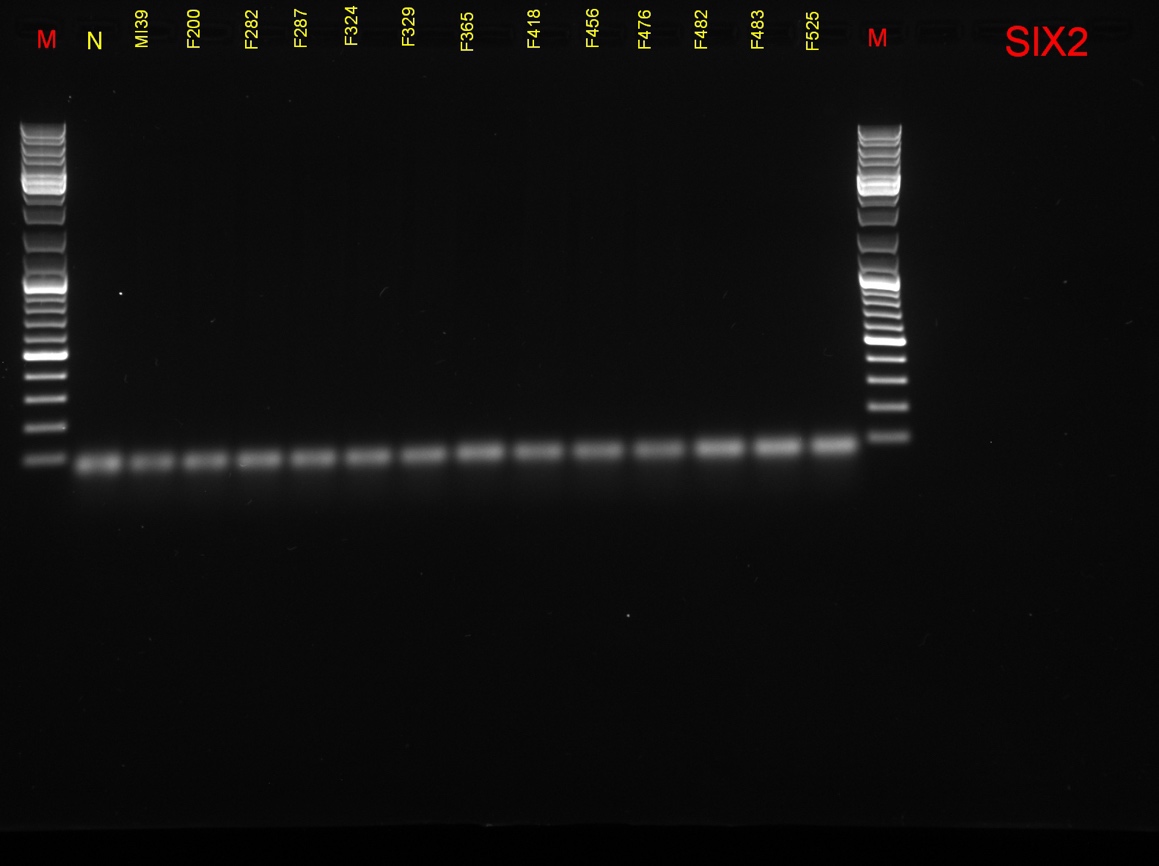


**Supplementary Figure 8.** Scan of the gel showing the absence of *SIX2* gene in the *Folini* isolates**.**

**
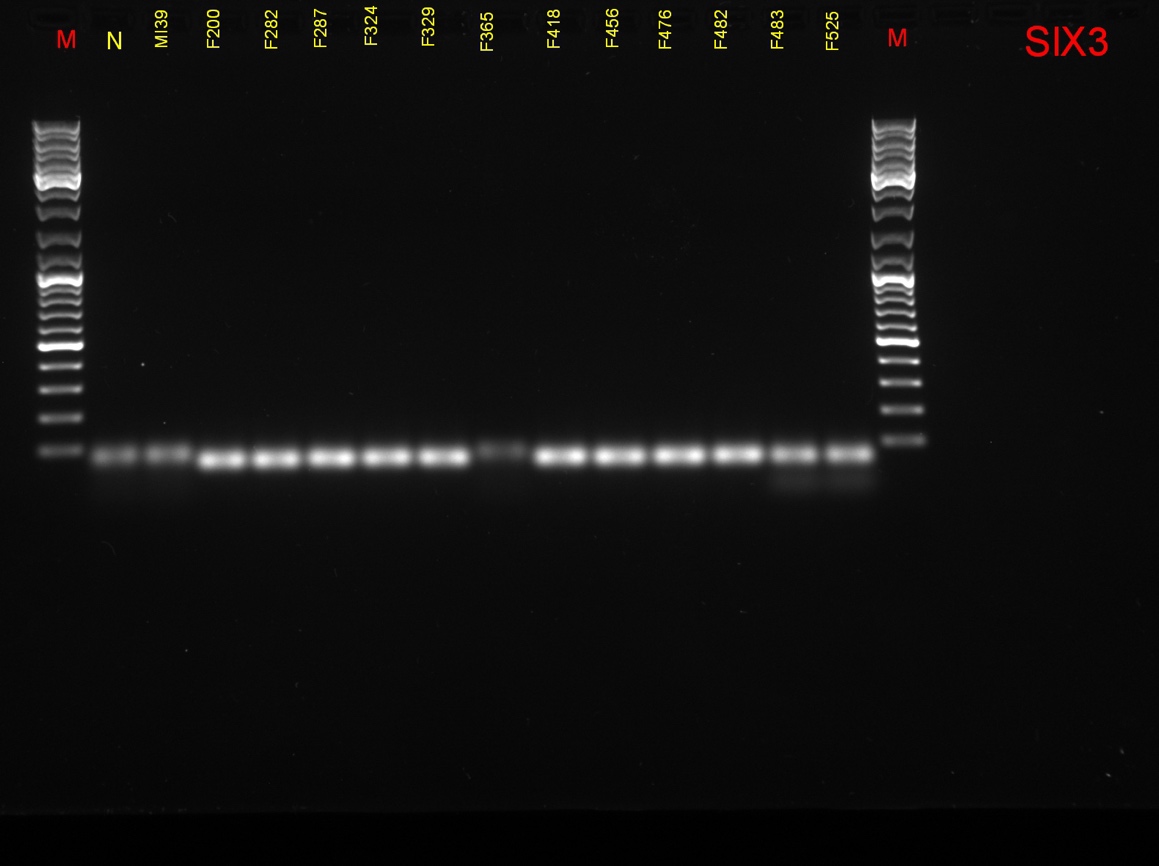
**

**Supplementary Figure 9.** Scan of the gel showing the absence of *SIX3* gene in the *Folini* isolates**.**

**
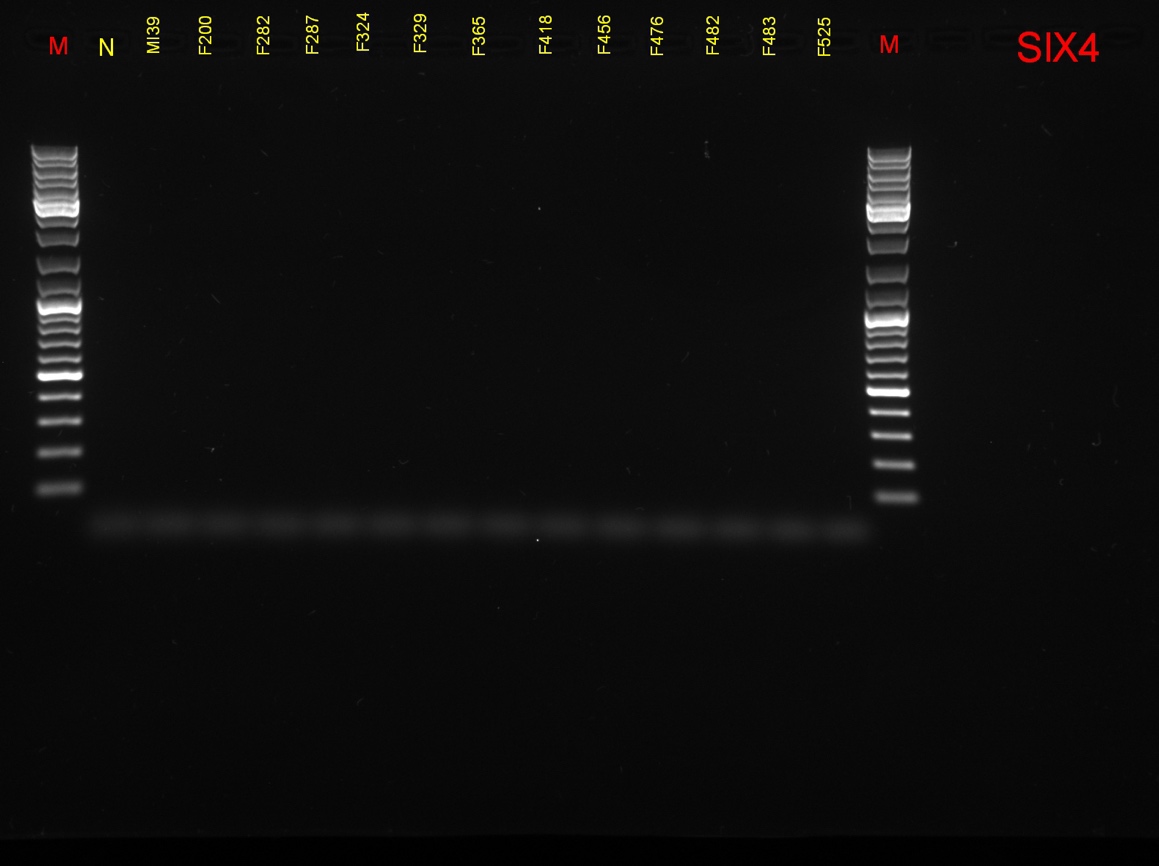
**

**Supplementary Figure 10.** Scan of the gel showing the absence of *SIX4* gene in the *Folini* isolates**.**

**
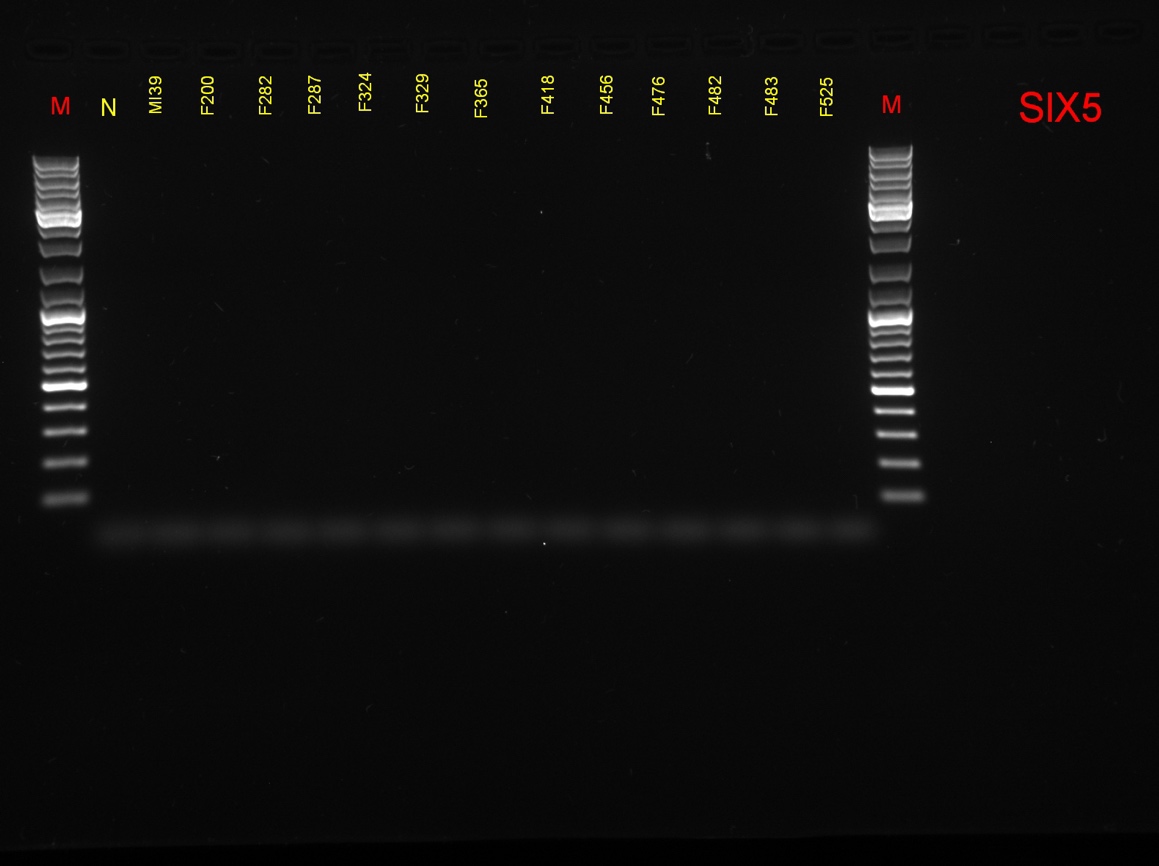
**

**Supplementary Figure 11.** Scan of the gel showing the absence of *SIX5* gene in the *Folini* isolates**.**

**
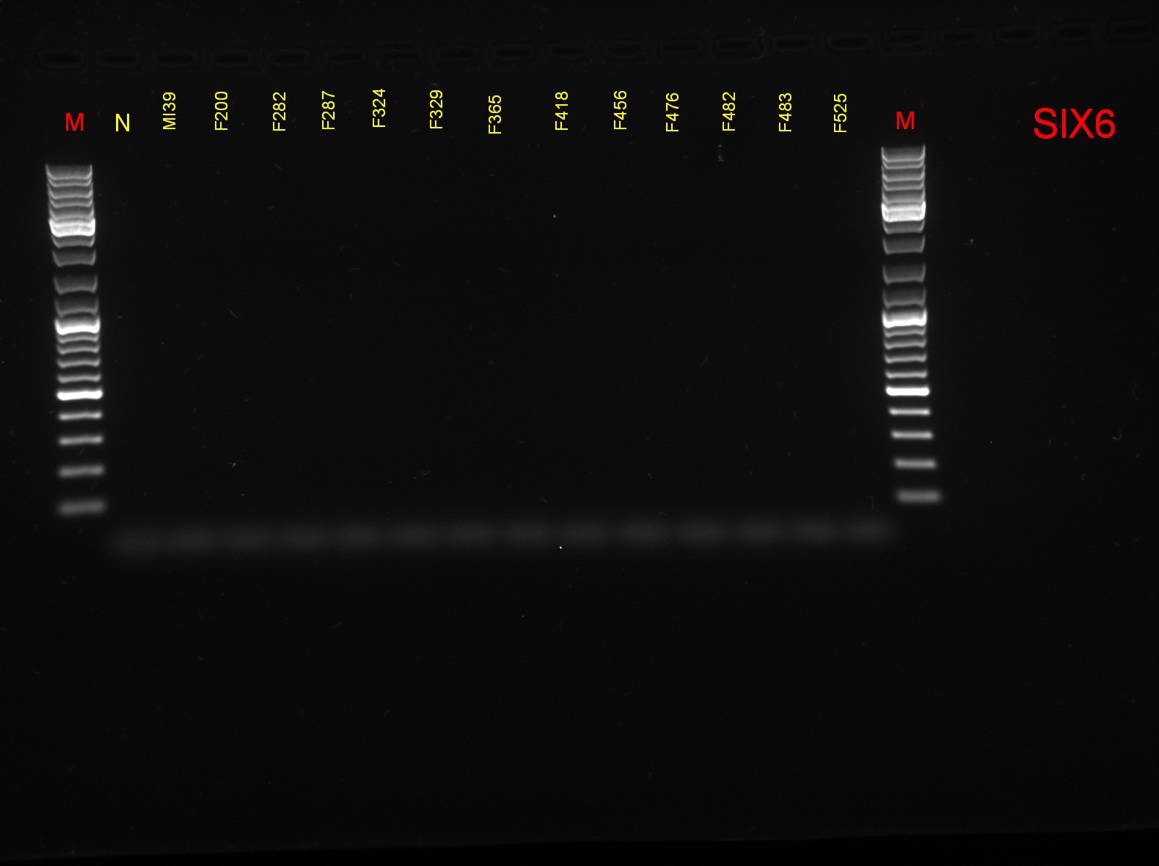
**

**Supplementary Figure 12.** Scan of the gel showing the absence of *SIX6* gene in the *Folini* isolates**.**

**
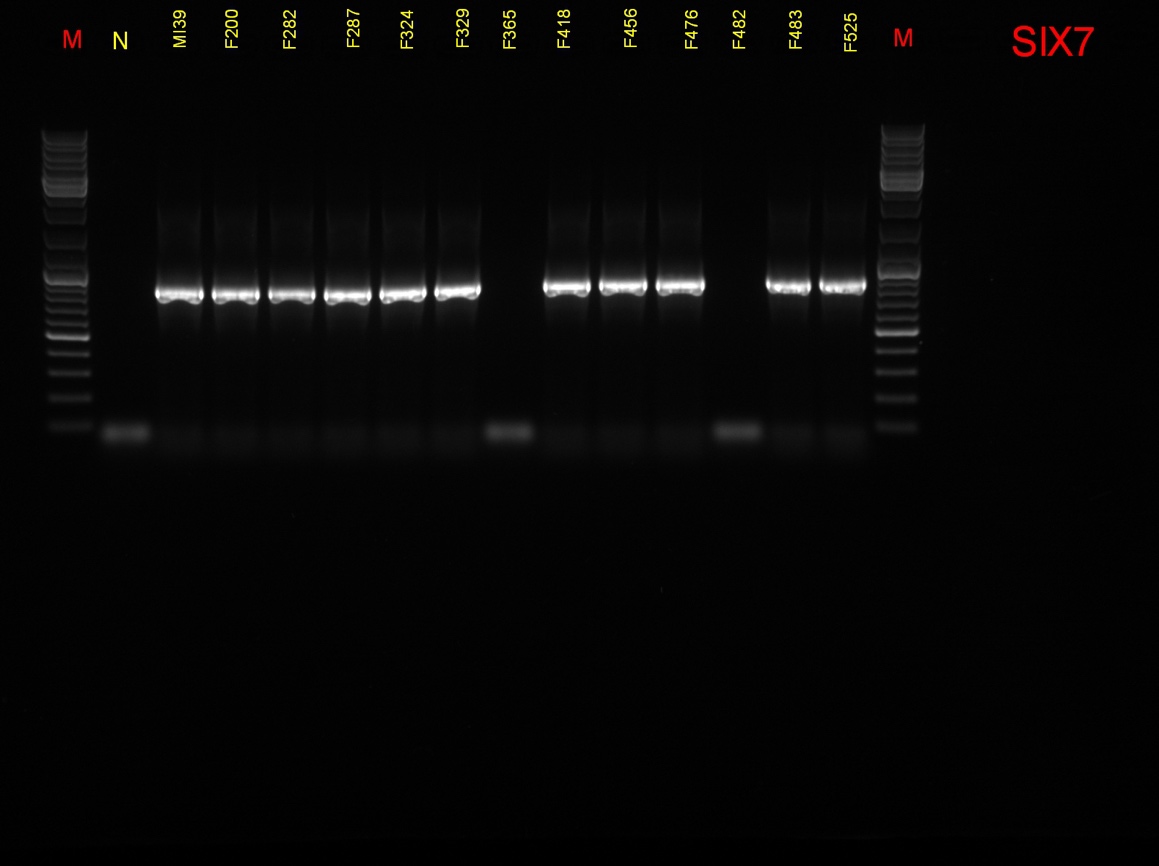
**

**Supplementary Figure 13.** Scan of the gel showing the presence of the *SIX7* gene orthologs in the *Folini* isolates**.**


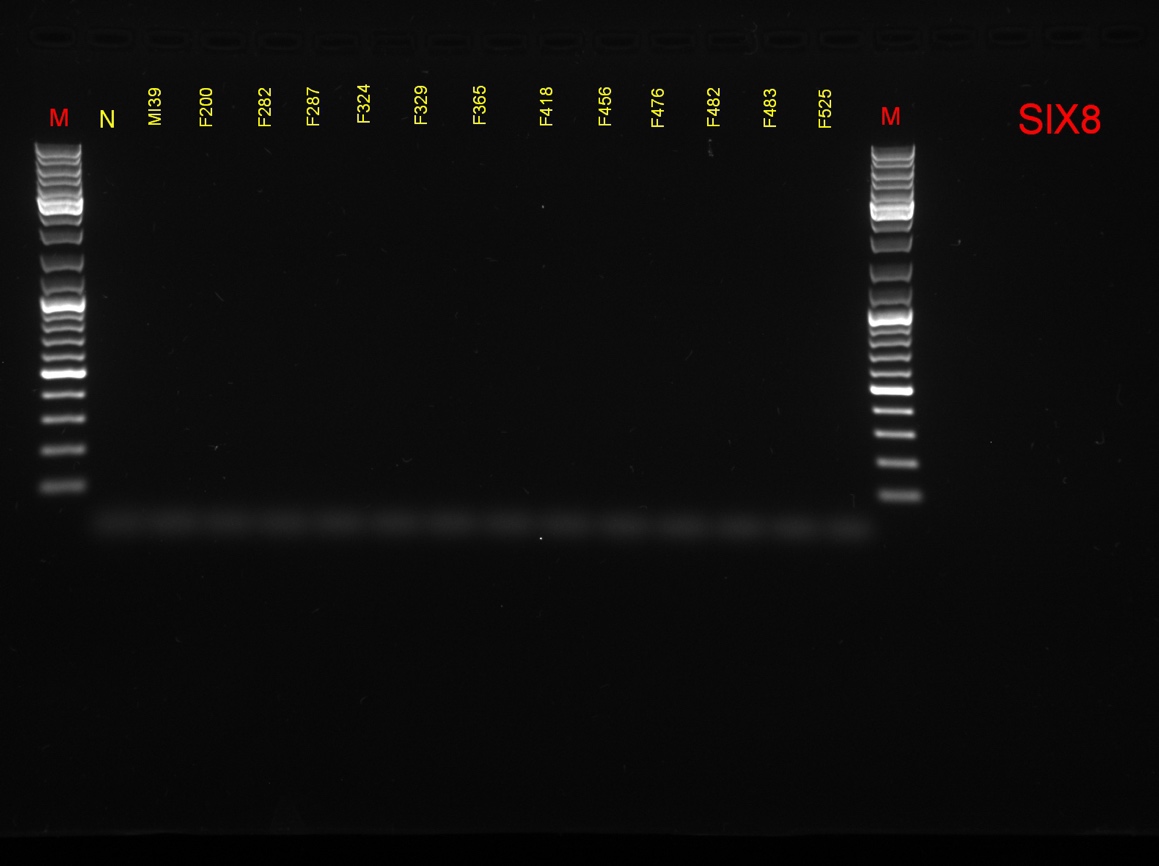


**Supplementary Figure 14.** Scan of the gel showing the absence of *SIX8* gene in the *Folini* isolates**.**


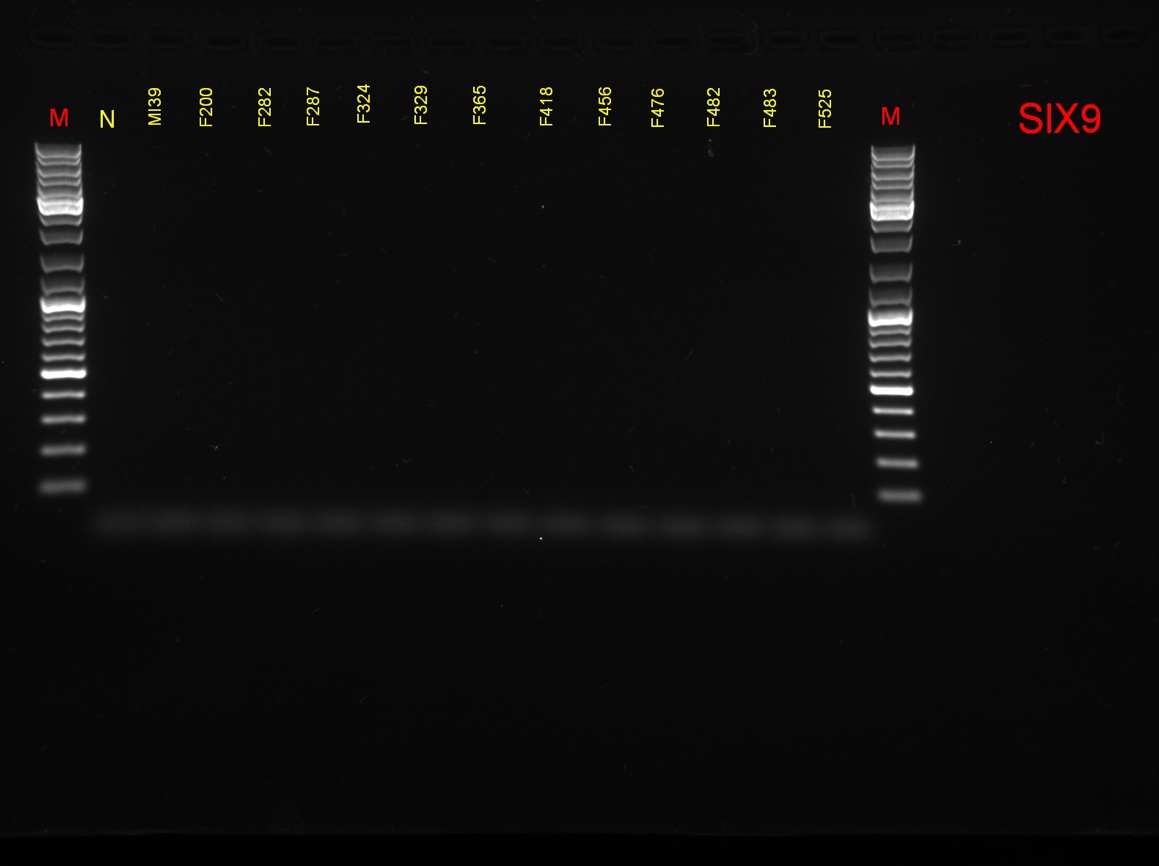


**Supplementary Figure 15.** Scan of the gel showing the absence of *SIX9* gene in the *Folini* isolates**.**


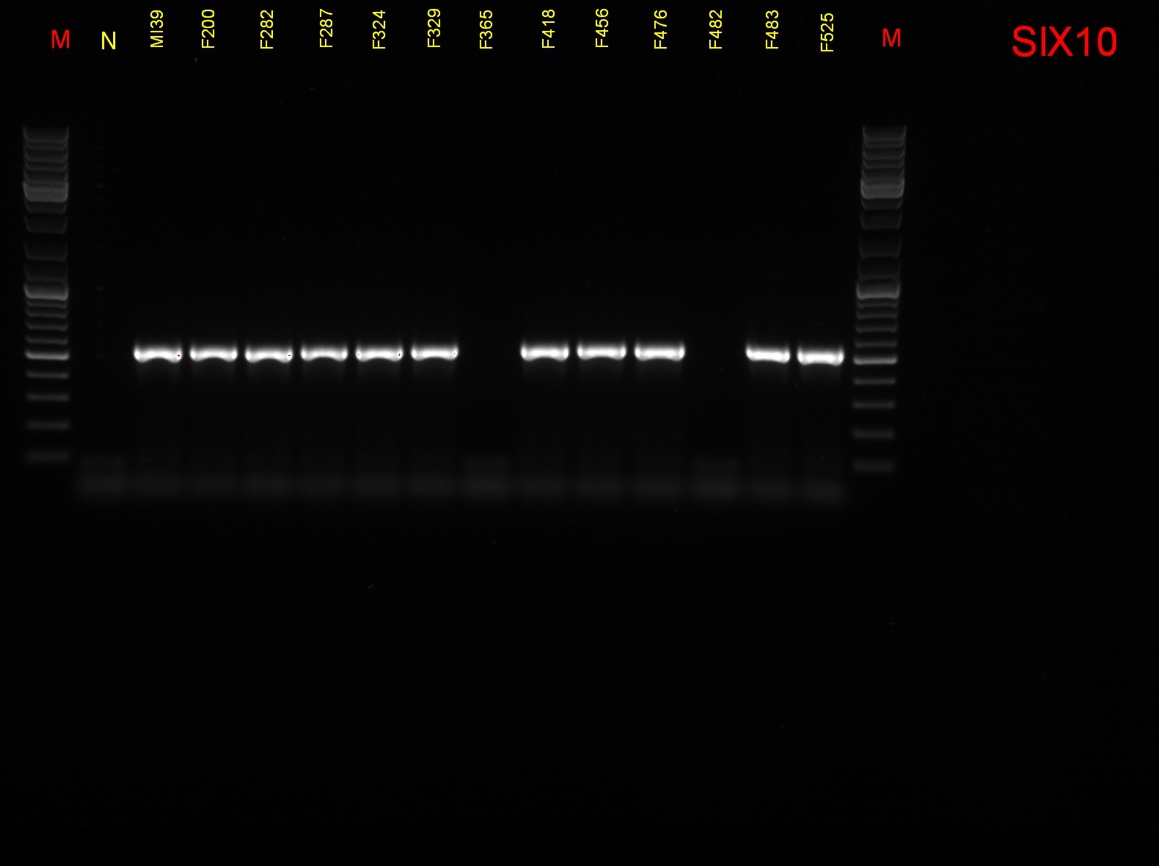


**Supplementary Figure 16.** Scan of the gel showing the presence of *SIX10* gene in the *Folini* isolates**.**


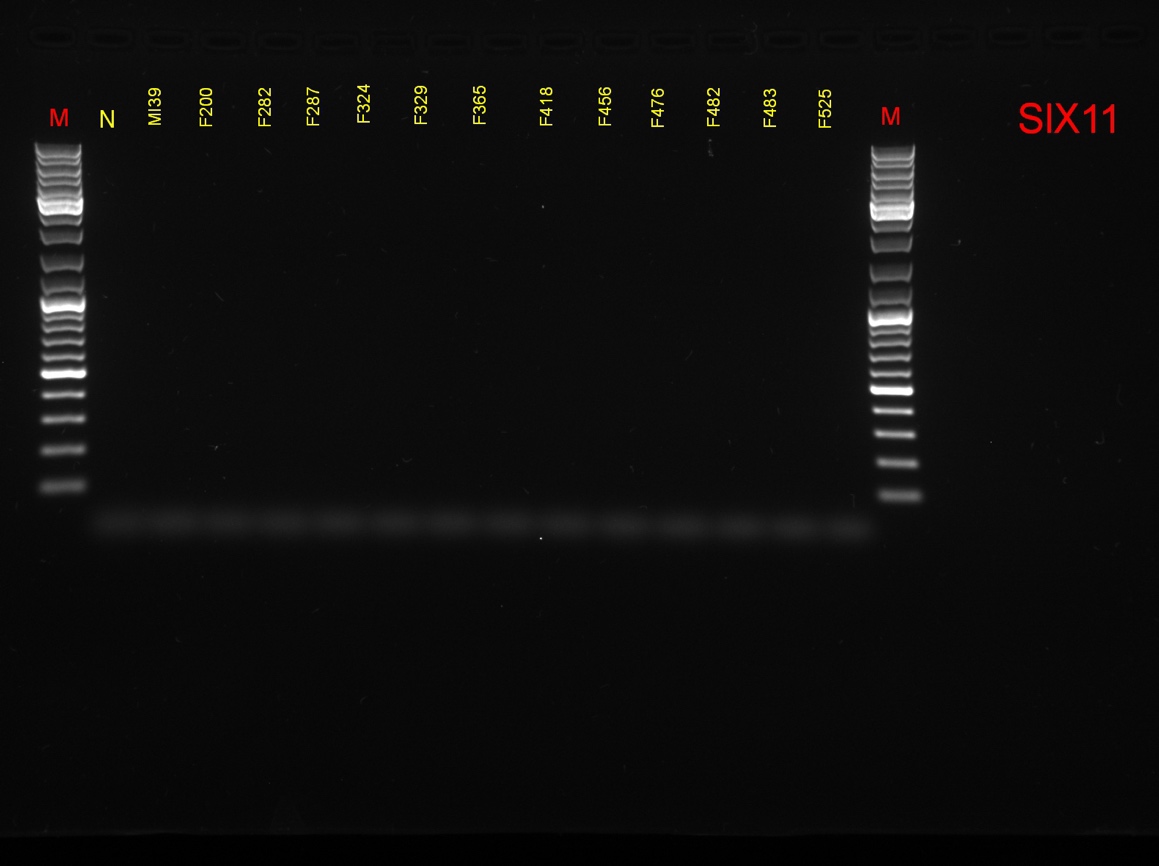


**Supplementary Figure 17.** Scan of the gel showing the absence of *SIX11* gene in the *Folini* isolates**.**


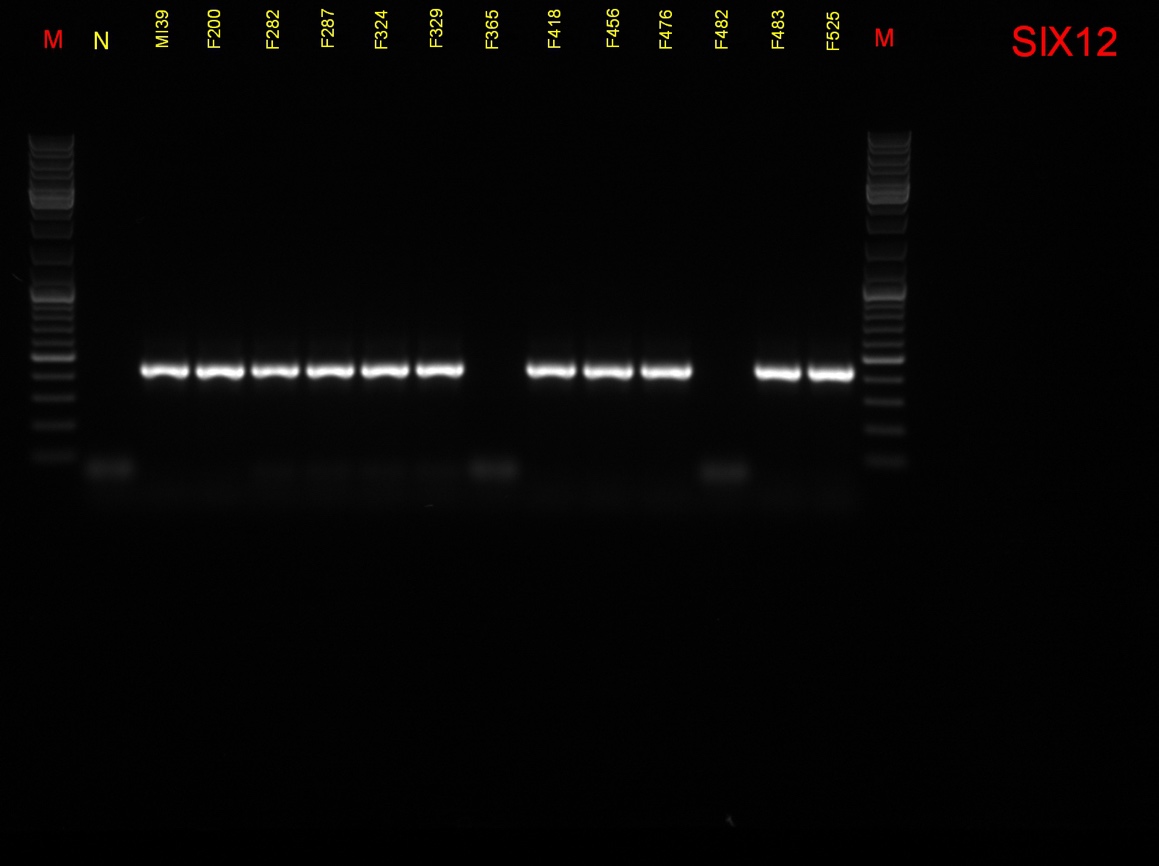


**Supplementary Figure 18.** Scan of the gel showing the presence of the *SIX12* gene orthologs in the *Folini* isolates**.**

**
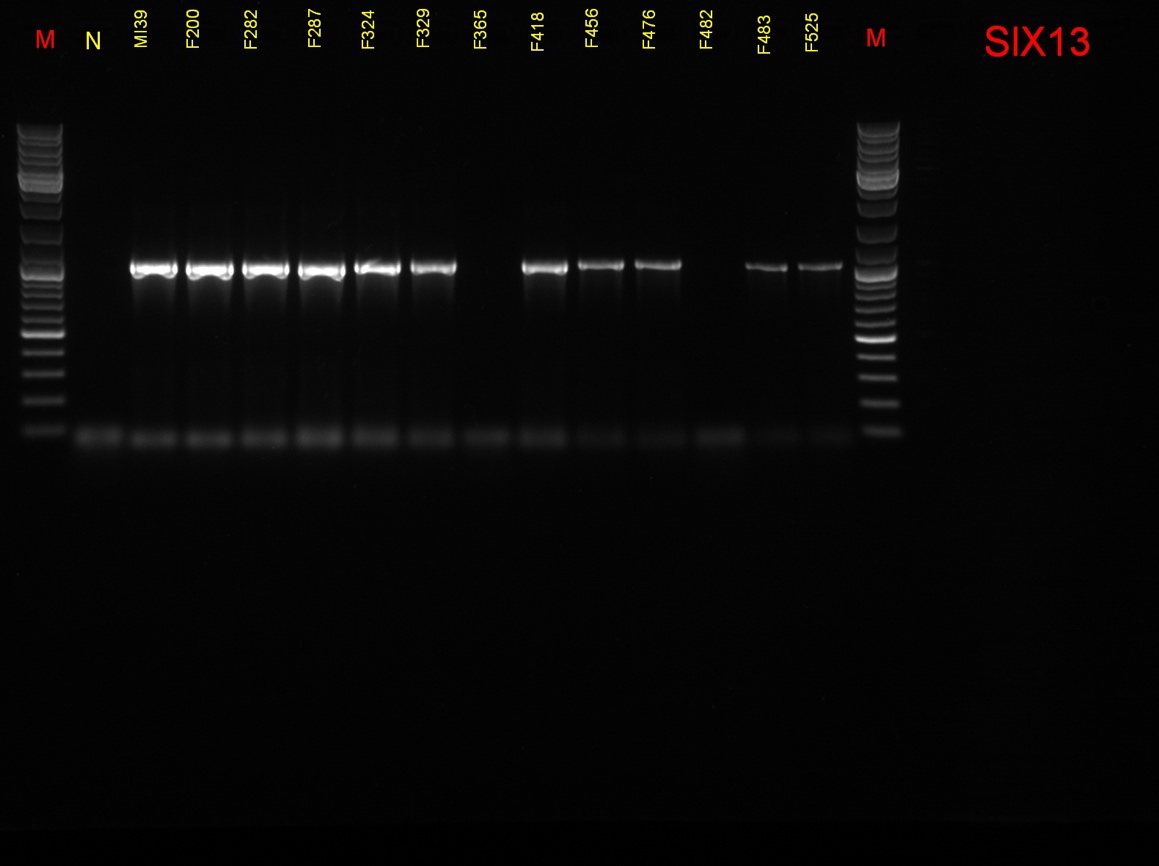
**

**Supplementary Figure 19.** Scan of the gel showing the presence of *SIX13* gene in the *Folini* isolates**.**

**
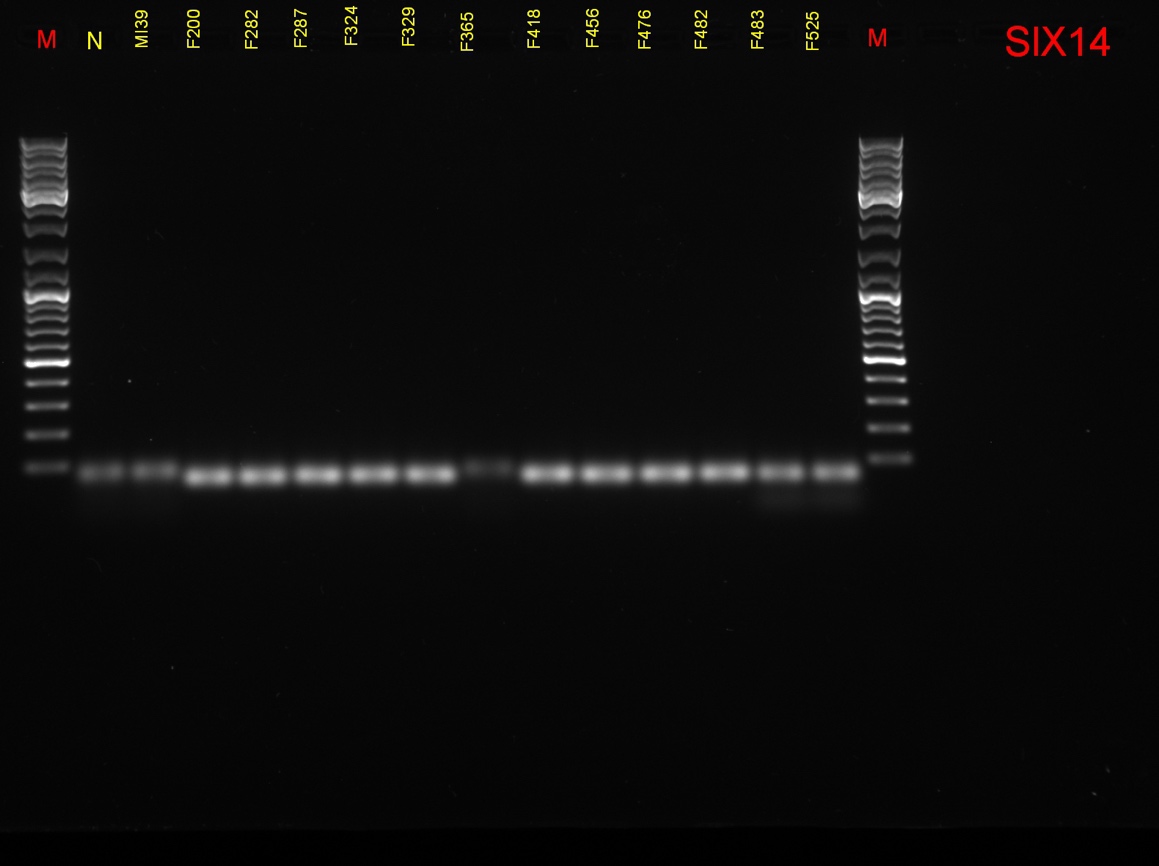
**

**Supplementary Figure 20.** Scan of the gel showing the absence of *SIX14* gene in the *Folini* isolates**.**
